# Single-Cell RNA Sequencing before and after Light Chain Escape Reveals Intrapatient Multiple Myeloma Subpopulations with Divergent Osteolytic Gene Expression

**DOI:** 10.1158/2767-9764.CRC-24-0170

**Published:** 2025-01-16

**Authors:** Denis Ohlstrom, Zachary J. Walker, Abhishek Pandey, Lorraine N. Davis, Krysta L. Engel, Zenggang Pan, Peter A. Forsberg, Tomer M. Mark, Austin E. Gillen, Daniel W. Sherbenou

**Affiliations:** 1 Division of Hematology, Department of Medicine, University of Colorado Anschutz Medical Campus, Aurora, Colorado.; 2 Biomedical Sciences and Biotechnology, Graduate School, University of Colorado Anschutz Medical Campus, Aurora, Colorado.; 3 Hematology-Oncology Fellowship Program, University of Colorado Anschutz Medical Campus, Aurora, Colorado.; 4 Department of Pathology, University of Colorado Anschutz Medical Campus, Aurora, Colorado.; 5 University of Colorado Cancer Center, University of Colorado Anschutz Medical Campus, Aurora, Colorado.

## Abstract

High-risk multiple myeloma is genomically unstable, comprising heterogeneous populations of tumor cells that evolve over time. Light chain escape (LCE) is a clinical phenomenon observed when light chains rise separately from M-spike values, which implies divergent tumor evolution. We sought to understand LCE by performing high-depth transcriptomic and phenotypic studies. We performed single-cell RNA-sequencing (scRNA-seq) and *ex vivo* drug sensitivity profiling on serial bone marrow biopsies from a patient with LCE at diagnosis, first relapse, and relapsed/refractory timepoints. scRNA-seq revealed distinct transcriptomic subpopulations with phenotypes that could be tracked separately by clinical serum light chain and M-spike values. Genes differentially expressed between subpopulations were assessed for generalizable effects on prognosis from the Multiple Myeloma Research Foundation CoMMpass and GSE24080 datasets. Notably, the LCE subpopulation exhibited gene expression profile featuring prominent *LAMP5* overexpression, which was associated with risk for osteolytic bone lesions. *Ex vivo* drug sensitivity testing displayed differential sensitivity of the subpopulations. Copy number variant inference showed that the transcriptomic subpopulation underlying LCE was related to a genetic subclone that evolved over time. Our findings illustrate that malignant subpopulations underly LCE in multiple myeloma. These studies imply that LCE and *LAMP5* gene overexpression portends for increased risk of osteolytic bone disease and adverse prognosis, findings that were confirmed in the subset of patients from the CoMMpass database with LCE.

**Significance:**

scRNA-seq was used to study a patient with high-risk multiple myeloma featuring LCE. LCE was rooted in a transcriptomic subpopulation that corresponded to a genetic subclone and established novel links between LCE and *LAMP5* overexpression to osteolysis and prognosis, validated in RNA-seq databases.

## Introduction

Although significant advances have been made in the treatment of multiple myeloma, some patients remain at risk for early mortality. With standard treatment including autologous stem cell transplant and immunomodulatory drug (IMiD) maintenance, the median survival now exceeds 10 years (1). However, high-risk subgroups have been recognized with much shorter survival (2, 3). One less understood feature of high-risk disease may be intrapatient tumoral heterogeneity. Light chain escape (LCE) is a clinical phenomenon that occurs in ∼10% of patients when isolated elevations in free light chain occur without detectable serum or urine monoclonal protein (medRxiv 2021.08.02.21261211v1; ref. 4). Some patients with LCE have been noted to have separate populations of cells expressing fully intact immunoglobulin and light chain only, suggesting that LCE may be a marker of subclonal evolution (5).

Recently, analysis of the Multiple Myeloma Research Foundation (MMRF) CoMMpass Study identified a total of 12 disease subtypes, of which proliferative, MAF bZIP transcription factor, and hyperdiploidy/p53 low subtypes had the shortest survival (medRxiv 2021.08.02.21261211v1). However, bulk mRNA sequencing could miss high-risk populations of cells, and tumor heterogeneity may influence patient outcomes. Comparative genomic hybridization studies have previously revealed a dynamic process of subclonal evolution and competition in multiple myeloma (6). Through next-generation DNA sequencing, essentially all patients have heterogeneous numbers of both clonal and subclonal genomic changes, even when first diagnosed (7). Recently, novel techniques such as single-cell RNA sequencing (scRNA-seq) have shed light on disease heterogeneity. The first such study discovered that transcriptomically distinct subpopulations were present in 34% (10/29) patients with newly diagnosed plasma cell neoplasms and that these were not explained by DNA mutation status alone (8). Interestingly, simultaneous sampling of bone marrow and lytic bone lesions showed to be related, but distinct subpopulation expression patterns between the two locations (9). Further use of scRNA-seq in different contexts will help understand intrapatient disease heterogeneity.

Here we performed an in-depth study of a patient with high-risk multiple myeloma and LCE with single-cell mRNA sequencing and *ex vivo* drug sensitivity profiling in serial samples through the course of their disease. In parallel, we also profiled the effects of anti-myeloma drugs on multiple myeloma cells using myeloma drug sensitivity testing (My-DST; ref. 10). In serial samples, distinct separate multiple myeloma cell subpopulations associated with LCE and full immunoglobulin heavy chain (IGH) production were found. Analysis of the differential gene expression of these subpopulations revealed how intrapatient heterogeneity influenced osteolysis and patient prognosis. These findings were extrapolated to large multiple myeloma datasets to show generalizable effects on prognosis and occurrence of osteolytic bone disease in multiple myeloma.

## Materials and Methods

### Sample acquisition

Bone marrow aspirates were collected at the University of Colorado Anschutz Medical Campus after informed consent and protocol approval. The study was performed following the guidelines of the Declaration of Helsinki. Mononuclear cells were isolated using SepMate Ficoll tubes (StemCell Technologies) and cryopreserved in Iscove Modified Dulbecco medium with 45% FBS and 10% DMSO as previously described (11).

### scRNA-seq

Bone marrow samples were thawed, resuspended in DNAse, and washed in PBS. A sort using a FACSAria (BD) was performed to remove dead cells. Single cells were captured using a Chromium X Series (10X Genomics) and sequenced on a NovaSeq6000 (Illumina). Feature-barcode matrix was produced from reads using Cellranger v.3.1.0 (10X Genomics). Data were analyzed using the Seurat package in R (12). Cells with low RNA detection (<250 genes) or high mitochondrial reads (>10% of all reads) were removed. Gene counts were normalized to the total number from each cell and natural log transformed. Variable genes used for clustering were identified using the M3Drop package (13). Cell clusters were visualized using the uniform manifold approximation and projection (UMAP) algorithm on the first 30 principal components from mitochondrial RNA regressed expression data. Doublets were removed using the DoubletFinder package (RRID:SCR_018771; ref. 14). Cell types were assigned using canonical markers as follows: T cells-*CD3*, B cells-CD20 (*MS4A1*), NK cells-*NKG7*, red blood cells-*HBB/HBA1*, megakaryocytes-*PPBP*, monocytes-*CD14*, and multiple myeloma-CD38/CD138 and *IGKC*. Differential expression was assessed between timepoints and multiple myeloma subpopulations. Gene-set enrichment was identified using the EnrichR package (RRID:SCR_001575) with Kyoto Encyclopedia of Genes and Genomes (KEGG) pathways (RRID: SCR_018771; refs. 15, 16). Copy number variant (CNV) inference based on the over- or underexpression of genes in continuous chromosomal locations was executed using the inferCNV package (bioRxiv 2021.10.18.463991v1). A more updated custom reanalysis was performed using STARsolo (https://github.com/alexdobin/STAR/blob/master/docs/STARsolo.md; ref. 17), and key findings did not change.

### Application of gene-sets to publicly available data

Subpopulation-specific gene-sets were defined by genes with an absolute fold change >2 and adjusted *P* value <0.05. Bulk gene expression and clinical data were obtained from the MMRF CoMMpass study (https://themmrf.org/we-are-curing-multiple-myeloma/mmrf-commpass-study/) and MAQC-II Project multiple myeloma dataset (GSE24080; ref. 18). IA13 TPM (transcripts per million) expression estimates from Salmon V7.2 were used for MMRF CoMMpass data. GSE24080 probes were annotated to the hgu133plus2 database; probes mapping to the same gene were averaged. Genes present in subpopulation gene-sets but not present in the publicly available datasets were not included in the analyses. For both datasets, gene-wise *z*-scores were calculated and summed to provide a gene-set score. Samples with a gene-set score >1 SD from the mean were considered high and <1 SD considered low. Overall survival (OS) was compared between high and low subtypes using the Survival package in R.

### *Ex vivo* drug sensitivity testing

To assess drug sensitivity, samples were subjected to myeloma drug sensitivity testing (My-DST) as described previously (10). In brief, thawed samples were resuspended in RPMI 1640 with l-glutamine containing 10% FBS, 100 U/mL penicillin, 100 μg/mL streptomycin (Thermo Fisher Scientific), and 2 ng/mL IL-6 (PeproTech) and treated for 48 hours. Agents tested in triplicate included bortezomib, carfilzomib, lenalidomide, pomalidomide, dexamethasone (Thermo Fisher Scientific), 4-hydroperoxy cyclophosphamide (Santa Cruz Biotechnology), and daratumumab (UC Health Pharmacy). To identify multiple myeloma cells, samples were stained with anti-CD38-PerCP-Cy5.5, anti-CD138-BV421, anti-CD45-BV510, anti-CD19-BB515, and anti-CD46-APC (BD Biosciences). Changes in viability were assessed by staining with LIVE/DEAD Fixable Near-IR Stain (Thermo Fisher Scientific). Samples were analyzed using a BD FACSCelesta equipped with a higher throughput sampler. Flow cytometry data were analyzed with FlowJo software (BD, RRID:SCR_008520).

### Histology and Ki-67 staining

For morphologic evaluation the bone marrow or tissue samples were formalin fixed and paraffin embedded and subsequently sectioned at 4.0 μm for hematoxylin and eosin staining. Immunohistochemical stains were performed using the Ventana Benchmark Ultra immunostainer (Ventana Medical Systems). Ki67 was utilized to assess the proliferation rate.

### Statistical analysis

Statistical significance was tested as follows (cut point of *P* < 0.05 for all comparisons): For scRNA-seq, *P* values were determined by logistic regression using FindMarkers in Seurat, with Bonferroni adjustment for multiple comparisons. For gene-set enrichment, hypergeometric test was used in the EnrichR package. For My-DST, *t* test was used for comparing cell viability between conditions. For osteolytic lesions, Fisher exact test was used for categorical data and *t* test for continuous data. For time to development of diffuse osteolytic lesions and OS, Cox proportional hazards model was used.

### Ethics approval statement

This study was performed following the guidelines of the Declaration of Helsinki. Written informed consent was obtained from all patients after Institutional Review Board approval.

### Data availability

The data generated in this study will be made publicly available in Gene Expression Omnibus (RRID:SCR_005012) with publication of the manuscript at GSE281459.

## Results

### scRNA-seq identifies malignant subpopulations with distinct transcriptomes

**Figure 1:**
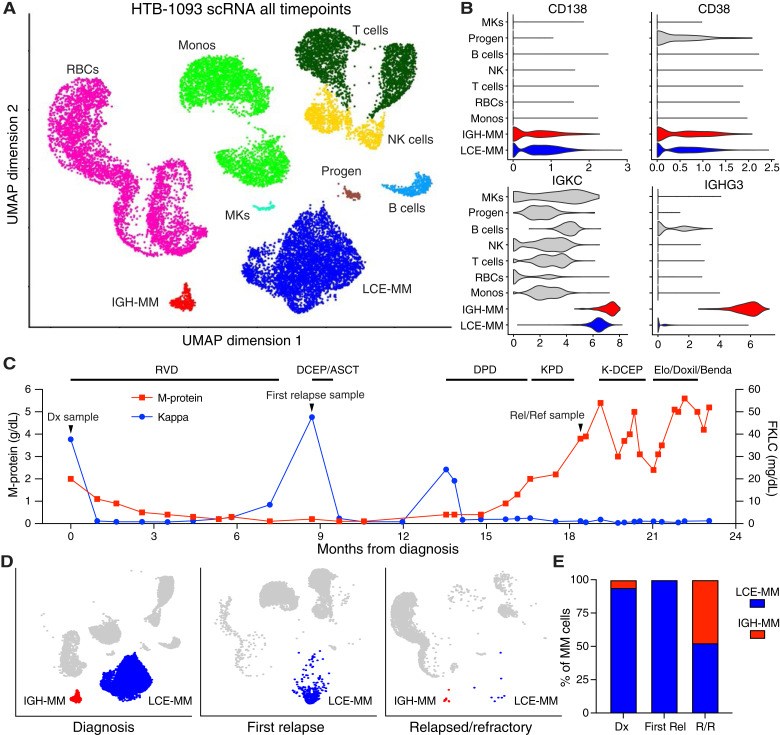
Patient with two multiple myeloma (MM) subpopulations correlating separately to monoclonal protein and free light chain detection through the course of disease. **A,** UMAP clustering analysis of bone marrow biopsy samples from patient #1093 at all three timepoints showed two distinct subpopulations of MM cells (LCE-MM and IGH-MM), along with the expected normal bone marrow cell types. **B,** Both MM subpopulations displayed transcript expression for the prototypical MM markers including high *CD138*, *CD38*, and light chain restricted to kappa only, but only IGH-MM expressed IGH. **C,** Clinical measurements of serum monoclonal (M) protein and free kappa light chain for patient #1093 from diagnosis through their disease course up to the date of transition to comfort care. **D,** UMAPs of scRNA-seq data from each timepoint individually, showing the two MM cluster (LCE-MM and IGH-MM) relative contributions from each sample. **E,** LCE-MM and IGH-MM were present at diagnosis, and only LCE-MM was present at first relapse. ASCT, autologous stem cell transplant; Benda, bendamustine; DCEP, dexamethasone, cyclophosphamide, etoposide, and cisplatin; DPD, daratumumab, pomalidomide, and dexamethasone; Elo, elotuzumab; FKLC, free kappa light chain; HTB, Hematology Tissue Bank; K, carfilzomib; KPD, carfilzomib, pomalidomide, and dexamethasone; MKs, megakaryocytes; Monos, monocytes; NK, natural killer; Progen, progenitors; RBCs, red blood cells; Rel, relapse; RVD, revlimid, velcade, and dexamethasone.

To investigate the biology of high-risk multiple myeloma, we used scRNA-seq in serial bone marrow biopsies from a 49-year-old male diagnosed with high-risk, R-ISS stage III, and R2-ISS stage IV multiple myeloma at our center. Cytogenetic analysis showed t(4;14), gain of chromosome 1q, loss of 13q, and a subclonal t(8;14; Supplementary Table S1). Despite aggressive treatment, this patient survived less than 2 years. Samples were obtained at diagnosis (sample #1093.1), first relapse when the patient’s clinical labs showed free LCE (sample #1093.2), and third relapse when the patient developed relapsed/refractory (R/R) disease (sample #1093.3). Live mononuclear cells were purified by FACS to remove dead cells. After quality control using the Seurat package in R (12), 17,629 of the 25,508 detected cell barcodes passed QC parameters: 9,426 from the diagnosis sample, 4,939 from first relapse sample, and 3,264 from the R/R sample. In aggregate analysis of all three timepoints, the patient displayed two distinct subpopulations by UMAP clustering, whereas normal cell types formed more discrete singlet or doublet clusters (Fig. 1A; “LCE-multiple myeloma” and “IGH-multiple myeloma”). LCE-multiple myeloma had restricted kappa light chain expression but did not have detectable *IGH* expression, whereas IGH-multiple myeloma expressed both IGH and a higher level of kappa light chain (Fig. 1B). Both subpopulations had prototypical multiple myeloma characteristics of high *CD138*, *CD38*, and clonally restricted kappa light chain expression. The remaining normal populations of lymphocytes, progenitors, etc., were identified by characteristic gene expression (Supplementary Fig. S1). Repeated analysis but with removal of *IGH* genes still showed two subpopulations (Supplementary Fig. S2A–S2D). The contrasting *IGH* expression suggested that only IGH-multiple myeloma would secrete fully intact antibody protein detectable by serum protein electrophoresis (SPEP), and LCE-multiple myeloma would only secrete kappa light chain and was ultimately responsible for free LCE.

Review of the patient’s clinical measurements of serum monoclonal (M) protein and free kappa light chain showed that elevation of their values alternated during the disease course, potentially coinciding with the two transcriptomic subpopulations (Fig. 1C). Both M-spike and kappa light chain decreased with induction treatment of lenalidomide/bortezomib/dexamethasone, culminating in a very good partial response. However, upon admission for postinduction autologous stem cell transplant, the patient was found to have LCE, with serum exhibiting only free kappa elevation and scRNA-seq detecting only the LCE-multiple myeloma cluster (Fig. 1D). The patient was re-induced with doxorubicin, cyclophosphamide, etoposide, and cisplatin and kept on high-dose melphalan and transplant. Unfortunately, the patient relapsed for a second time just 3 months after transplant, again showing isolated kappa elevation. The patient regained response with a third line consisting of daratumumab, pomalidomide, and dexamethasone, but relapsed again shortly after. Surprisingly, this third relapse showed only M-spike elevation by clinical labs. The sample obtained at the third timepoint was unfortunately limited an apparent processing issue with low final cell number submitted for scRNA-seq, but seemed to detect cells from both subpopulations (Fig. 1D and E). Thereafter, the patient’s disease was not controlled, with M-spike remaining high despite multiple lines of therapy. Bone marrow biopsy of the R/R disease was obtained after progressing through multiple lines of attempted therapy. Unfortunately, the disease was refractory and disease-directed treatment was discontinued.

**Figure 2:**
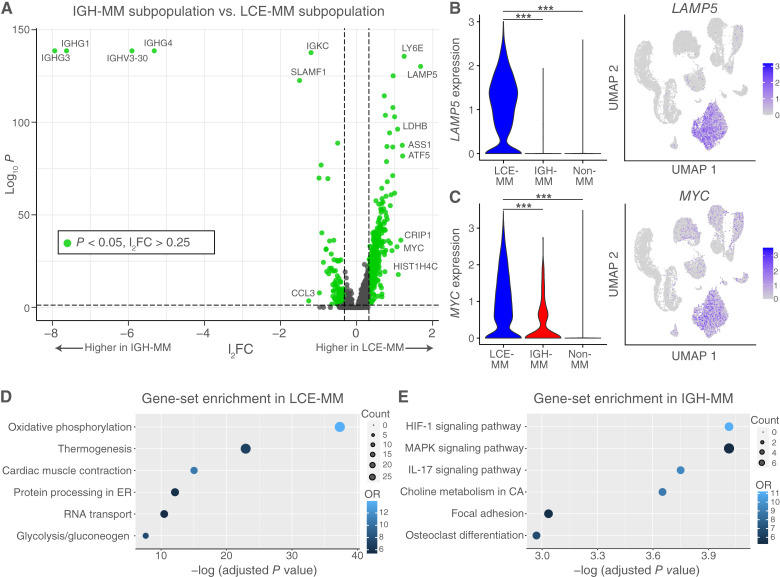
Differential gene expression between multiple myeloma (MM) subpopulations at diagnosis. **A,** Volcano plot showing genes expressed at higher levels at timepoint 1 in LCE-MM and those higher expressed in IGH-MM with log_2_ fold-change (l_2_FC) cutoff above 0.25 (vertical dashed lines) and statistical significance defined as multiple comparison adjusted *P* value < 0.05 (horizontal dashed line) by linear regression (LCE-MM *n* = 4,370 cells, IGH-MM *n* = 281 cells). **B,***LAMP5* had the highest fold change in LCE-MM compared with IGH-MM (l_2_FC: 1.68, *P* = 6.68 × 10^−131^, left), with an expression pattern mostly restricted to this subpopulation and low in normal cell types (right). **C,** Another notable transcript overexpressed in LCE-MM compared with IGH-MM was the oncogene *MYC* (l_2_FC: 1.06, *P*: 1.63 × 10^−33^). Of note, both MM subpopulation overexpressed *MYC* compared with normal cells. **D,** Gene-set enrichment analysis using the KEGG identified the oxidative phosphorylation pathway to be upregulated in LCE-MM. **E,** In IGH-MM, the HIF-1 signaling pathway was the most overrepresented gene-set. CA, cancer; ER, endoplasmic reticulum; OR, odds ratio (***, *P* < 0.001).

As the multiple myeloma cell production of either kappa light chain or M-spike was associated with different clinical behavior, we compared gene expression of the two malignant subpopulations at the diagnosis timepoint to understand their distinct biology. Differential expression between the subpopulations at diagnosis identified 579 genes that were higher in LCE-multiple myeloma and 127 genes higher in IGH-multiple myeloma, with significance defined as multiple comparison adjusted *P* value < 0.05 with a log_2_ fold-change (l_2_FC) > 0.25 (Fig. 2A; Supplementary Tables S2 and S3). *LAMP5* was the specifically overexpressed just in LCE-multiple myeloma (Fig. 2B). *MYC* was also overexpressed in LCE-multiple myeloma more than its expression in IGH-multiple myeloma, potentially related to the subclonal t(8;14) observed upon diagnosis by clinical FISH (Fig. 2C). Another notable transcript was *FABP5*, a prominent gene in the gene expression profile (GEP) 70 and GEP5 scores for high-risk gene expression (Supplementary Table S2; refs. 19, 20). Immunoglobulin heavy chain genes comprised the top overexpressed genes in IGH-multiple myeloma (Supplementary Fig. S3). Gene-set enrichment analysis identified the oxidative phosphorylation pathway and hypoxia-inducible factor-1 (HIF-1) signaling pathway as the most overrepresented from the genes upregulated in LCE-multiple myeloma and IGH-multiple myeloma, respectively (Fig. 2D and E). Twenty-four genes from the oxidative phosphorylation pathway were significantly higher in LCE-multiple myeloma compared with IGH-multiple myeloma, and 23/24 were also significantly higher compared with normal cells (Supplementary Fig. S4). Five genes from the HIF-1 signaling pathway were significantly higher in IGH-multiple myeloma compared with LCE-multiple myeloma, with 3/5 of the transcripts also being significantly higher compared with normal cells (Supplementary Fig. S5). Based on these analyses, LCE-multiple myeloma and IGH-multiple myeloma showed different gene expression and active biologic pathways, suggesting that transcriptomic changes contributed to their distinct clustering and behavior.

### Distinct drug sensitivity profiles in multiple myeloma subpopulations

**Figure 3:**
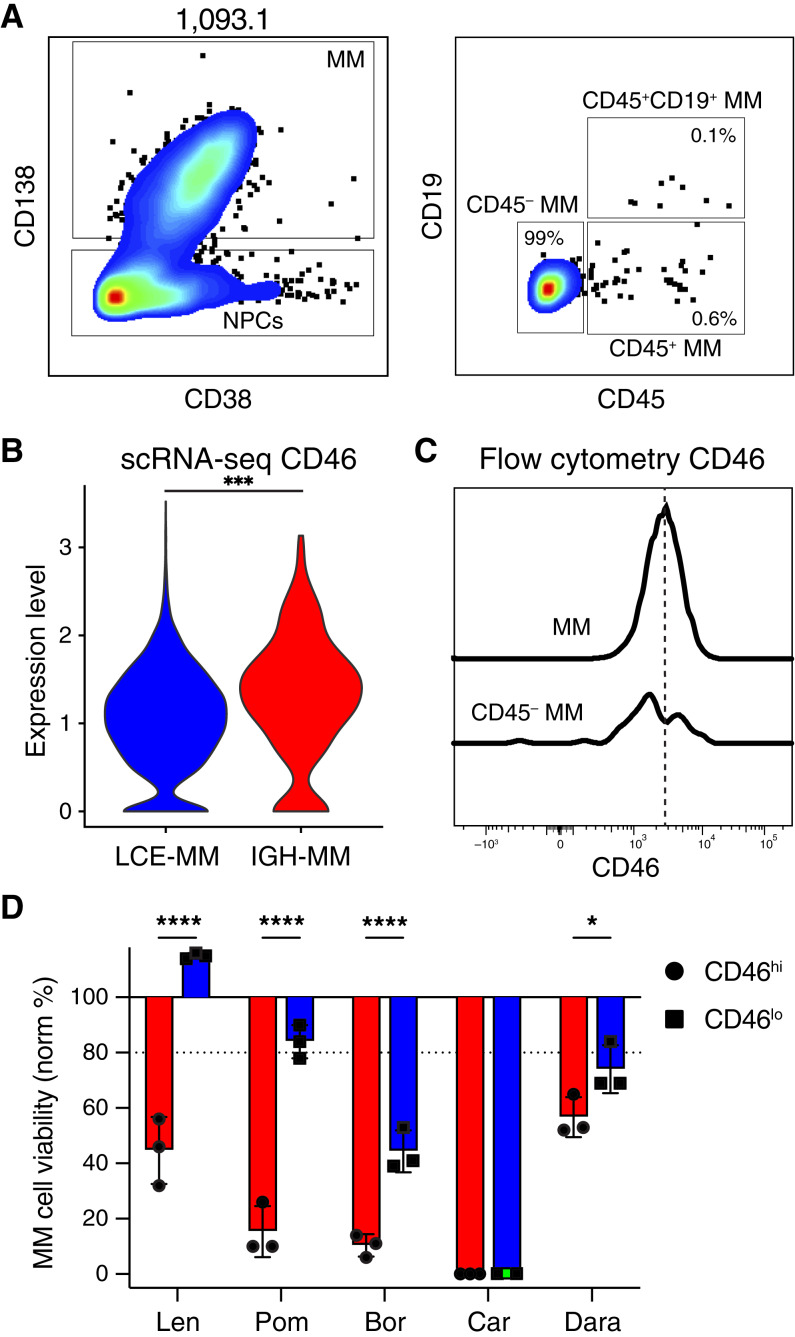
Differential myeloma drug sensitivity profiles of malignant subpopulations at diagnosis. **A,** Flow cytometry showed a monotypic population by typical markers CD138 and CD38 (left), but showed rarer subpopulations with CD45 expression, with or without CD19 (right) that were not detected by scRNA-seq. **B,** Of the cell surface proteins measured by flow cytometry, the mRNA for CD46 showed the most differential expression (l_2_FC = 0.44, *P* = 5.76 × 10^−7^) between the LCE-multiple myeloma (MM) and IGH-MM subpopulations. **C,** Relatively CD46-high and CD46-low populations were divided to approximate subpopulation drug sensitivity of IGH-MM and LCE-MM, respectively. **D,** The drug sensitivity profiles implied that the CD46-high/IGH-MM subpopulation had a primary IMiD refractory phenotype and was relatively less bortezomib sensitive compared with LCE-MM (*n* = 3, tested by *t* test, bars depict standard deviation. *, *P* < 0.05; ***, *P* < 0.001). Bor, bortezomib; Car, carfilzomib; Dara, daratumumab; Len, lenalidomide; norm %, percent viability normalized to untreated controls; Pom, pomalidomide.

To more deeply understand the poor prognosis of this patient and evaluate the connection between phenotype and gene expression, we performed flow cytometry and drug sensitivity testing. Previously, we developed a platform termed My-DST, which measures loss of viability specifically in multiple myeloma cells to clinically used drug treatments in short-term 48-hour cultures (10). This approach utilizes flow cytometry with markers variably expressed in the patient’s subpopulations, enabling the comparison of drug sensitivity between IGH-multiple myeloma and LCE-multiple myeloma. Unselected mononuclear cells from the diagnosis sample were screened at drug concentrations established to be efficacious in My-DST: 10 μmol/L lenalidomide, 10 μmol/L pomalidomide, 2.5 nmol/L bortezomib, 2.5 nmol/L carfilzomib, and 10 nmol/L daratumumab, with each condition performed in triplicate. Multiple myeloma cells were identified by CD38 and CD138 expression (Fig. 3A). *CD46* was identified as the most differentially expressed marker in our flow cytometry panel, as it had higher expression in IGH-multiple myeloma (l_2_FC = 0.44, *P* = 5.76 × 10^−7^; Fig. 3B). By flow cytometry, CD45-negative multiple myeloma cells had bimodal CD46 expression, which was used to stratify all multiple myeloma cells into high or low CD46 (Fig. 3C). The change in the number of multiple myeloma cells with high or low CD46 were measured in each drug treatment well relative to matched populations in untreated wells. Considering our previous experience with the My-DST protocol, we have established a working cutoff of 80% multiple myeloma cell viability compared with untreated controls to distinguish clinically predictive sensitivity or resistance (10). The CD46 low cells, roughly equated to the LCE-multiple myeloma subpopulations, showed intrinsic resistance to the IMiDs, but remained sensitive to proteosome inhibitors and daratumumab (Fig. 3D). The expression of CD38 in LCE-multiple myeloma was present but slightly lower than in IGH-multiple myeloma (see Fig. 1B), corresponding with the slightly less (but statistically significant) response to daratumumab *ex vivo*. Thus, we concluded that the LCE-multiple myeloma cells harbored more inherent drug resistance at diagnosis relative to the IGH-multiple myeloma subpopulation.

### LCE-specific gene expression linked to osteolytic bone disease and poor prognosis

**Figure 4:**
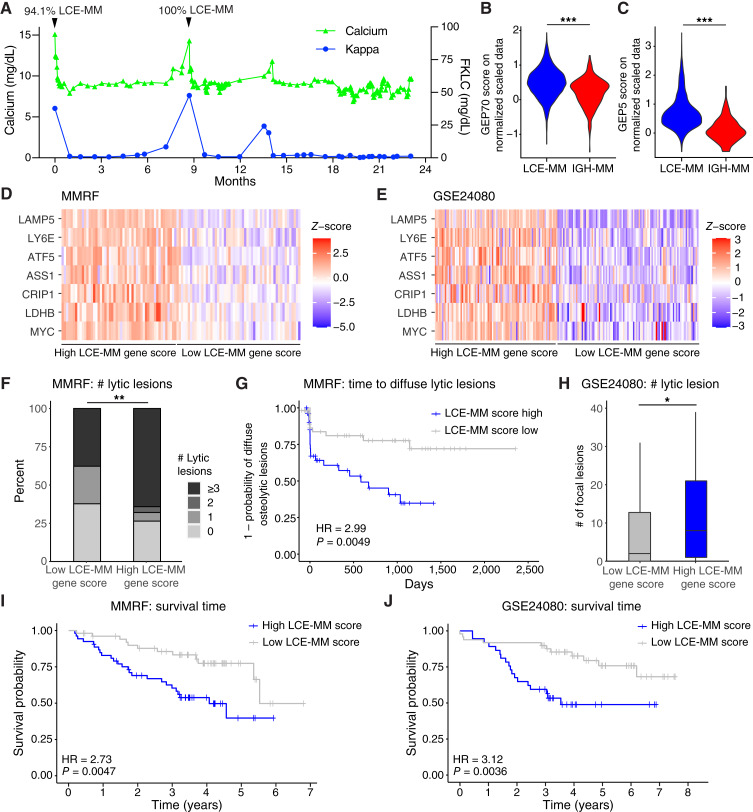
Multiple myeloma (MM) subpopulation gene-set exhibits osteolytic features and is detrimental to OS. **A,** Through the disease course for patient #1093, spikes in serum calcium occurred during periods of disease activity with high levels of LCE-MM. **B,** The GEP70 score for high-risk MM was significantly higher in the LCE-MM subpopulation compared with the IGH-MM subpopulation. **C,** Five key genes in the GEP70 score were even more upregulated in LCE-MM compared with IGH-MM. **D,** In the MMRF cohort, 325 patients had RNA-seq and osteolytic data at diagnosis, with 83 showing high LCE-MM gene-set scores and 79 showing low LCE-MM gene-set scores by gene-set enrichment analysis. **E,** In the GSE24080 cohort, 254 microarray samples were available from patients at diagnosis, with gene-set enrichment identifying 57 new diagnosis samples with high and 62 samples with low LCE-MM gene-set scores. **F,** Most of the samples in the MMRF dataset that were enriched for the LCE-MM signature had ≥3 osteolytic lesions (*, *P* < 0.05; **, *P* < 0.01; ***, *P* < 0.001 by the Fisher exact test). **G,** LCE-MM enriched samples in the MMRF developed diffuse osteolytic lesions at a more rapid rate than those with a low LCE-MM signature (*P* = 4.9 × 10^−3^ by Cox-p test). **H,** Samples in the GSE24080 dataset that were enriched for the LCE-MM gene-set had increased lytic bone lesions relative to samples with a low LCE-MM gene-set score (*P* < 0.05 by *t* test). **I,** In the MMRF dataset patients with high LCE-MM gene enrichment score had worse OS than those with low LCE-MM gene enrichment. **J,** In the GSE24080 dataset patients with high LCE-MM gene enrichment score also had worse OS than those with low LCE-MM gene enrichment (log-rank test). Dotted lines indicated 50% survival.

Whereas the IGH-multiple myeloma gene signature was most notable for elevated IGH, the LCE-multiple myeloma signature contained biologically intriguing transcript elevations including *LAMP5* and *MYC*. *MYC* has long been appreciated as an important oncogene in multiple myeloma, whereas scRNA-seq studies have implicated *LAMP5* to be a novel overexpressed gene in multiple myeloma (8, 9, 21). Interestingly, localized *LAMP5* overexpression has been reported in osteolytic lesions, one of the most debilitating complications of multiple myeloma (22). As *LAMP5* was overexpressed in LCE-multiple myeloma, we hypothesized that this cell subpopulation may have been prone to cause lytic bone disease in our patient. LCE-multiple myeloma was the predominant multiple myeloma subtype at diagnosis and first relapse. Clinical calcium measurements spiked at exactly when the LCE-multiple myeloma was predominant at timepoints 1 and 2 as detected by scRNA-seq, and when light chain was highest as measured clinically (Fig. 4A). Thus, the times in which the *LAMP5*-high subpopulation was prevalent corresponded to calcium spikes, implying a direct link between the features of LCE-multiple myeloma and osteolysis. Previous gene expression signature scores have been associated with high-risk disease in patients with multiple myeloma, such as the GEP70 (19). We applied this to the subpopulations in our patient and found the GEP70 score to be significantly elevated (Fig. 4B). A subset of five key genes from the GEP70 score (*ENO1*, *FABP5*, *TRIP13*, *TAGLN2*, and *RFC4*; ref. 20) was even more enriched (Fig. 4C). Therefore, the gene expression in the LCE-multiple myeloma subpopulation also seemed to confer a poor prognosis.

To evaluate the generalizability of the link between *LAMP5* and osteolysis in patients with multiple myeloma, we compared the LCE-multiple myeloma gene signature in larger cohorts of patients. We identified samples with similar transcriptome signatures in the MMRF bulk RNA-seq and the GSE24080 microarray datasets. The gene-set for LCE-multiple myeloma comprised genes with l_2_FC > 1.0 and *P* < 0.05 over IGH-multiple myeloma in the scRNA-seq data (labeled LCE-multiple myeloma high genes in Fig. 2A). These LCE-multiple myeloma high genes were specific to multiple myeloma cells with low or no detectable expression in normal cells (Supplementary Fig. S6A). In the MMRF database, gene-set enrichment identified 82 (25%, 82/325) of new diagnosis samples to have high LCE-multiple myeloma gene-set scores and 24% (79/325) to have low LCE-multiple myeloma gene-set scores (Fig. 4D). In the GSE24080 dataset, 22% (57/254) had high LCE-multiple myeloma gene-set enrichment scores and 24% (62/254) had low scores (Fig. 4E). The MMRF database codified osteolytic lesions as 0, 1, 2, and ≥3 as well as the number of days to development of “diffuse osteolytic lesions.” Of samples enriched for the LCE-multiple myeloma transcriptome, 64% had ≥3 osteolytic lesions compared with 37% in LCE-multiple myeloma low samples (*P* = 3 × 10^−3^, Fig. 4F). In addition, LCE-multiple myeloma gene-set–enriched samples were labeled in the MMRF database at their time to develop diffuse osteolytic lesions with a HR of 2.99 [confidence interval (CI), 6.4–1.4; *P* = 4.9 × 10^−3^, Fig. 4G]. Similarly, samples in GSE24080 enriched for the LCE-multiple myeloma gene-set had a 1.5-fold increase (*P* = 3.74 × 10^−2^) of osteolytic lesions relative to samples with a low LCE-multiple myeloma gene-set score (Fig. 4H). *LAMP5* alone had a statistically significant adverse effect on survival in the MMRF database (HR = 1.96; *P* = 0.0047), but a nonsignificant impact on the development of diffuse osteolytic lesions (HR = 1.3; *P* = 0.45; Supplementary Fig. S6B and S6C). Interestingly, we found that *LAMP5* knockdown did not affect myeloma cell viability or induce apoptosis in cell lines (Supplementary Fig. S6D and S6E). Thus, approximately one fourth of patients diagnosed with multiple myeloma have gene expression profiles similar to the LCE-multiple myeloma subpopulation and display a predilection for lytic bone disease as a disease-related complication.

Next, we evaluated whether *LAMP5* and the other most overexpressed genes in the subclonal LCE-multiple myeloma subpopulation affected prognosis on their own. The gene signature of LCE-multiple myeloma seemed high risk in both the MMRF and GSE24080 datasets. Patients with high enrichment scores for the LCE-multiple myeloma gene-set in MMRF had median OS of 4.07 versus 5.52 years with a hazard ratio of 2.73 (CI, 5.5–1.4; *P* = 4.72 × 10^−3^; Fig. 4I). In GSE24080 the median OS was 3.57 years in the LCE-multiple myeloma high group, LCE-multiple myeloma low had 68.1% of patients surviving at 6.7 years (max follow up in this cohort) with a hazard ratio of 3.12 (CI, 6.7–1.4; *P* = 3.65 × 10^−3^; Fig. 4J). The gene signature in IGH-multiple myeloma was dominated by high expression Ig heavy chain genes (Supplementary Fig. S7A and S7B). The IGH-multiple myeloma gene signature showed no difference in the MMRF dataset and a better outcome in the GSE24080 dataset (Supplementary Fig. S7C and S7D). The reason for this difference between datasets is not clear. Overall, expansion of the LCE-multiple myeloma subpopulation in our patient led to their shortened survival, which is mirrored in larger cohorts in which transcriptionally similar patients who also had inferior survival.

**Figure 5:**
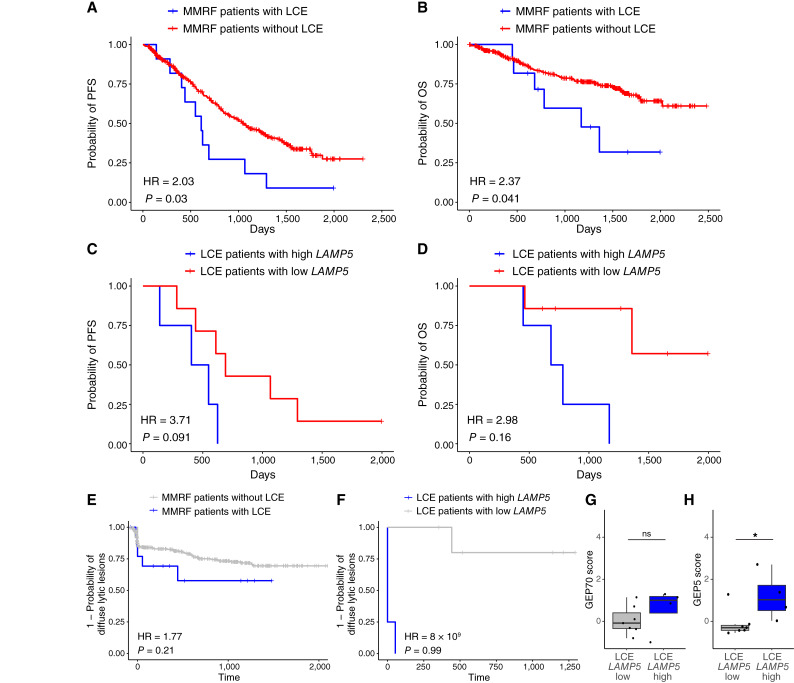
Patients from the CoMMpass database with LCE have a poor prognosis. **A,** Fifteen patients identified from CoMMpass with LCE showed significantly shorter PFS from diagnosis than those without LCE. **B,** Patients identified from CoMMpass with LCE showed significantly shorter OS from diagnosis than those without LCE. **C,** Patients from CoMMpass with LCE with high *LAMP5* expression had a trend toward significantly shorter PFS compared with those with LCE and low *LAMP5*. **D,** Patients from CoMMpass with LCE with high *LAMP5* expression had nonsignificantly shorter OS compared with those with LCE and low *LAMP5*. **E,** Patients from CoMMpass with LCE had nonsignificantly shorter time to diffuse lytic lesions compared with those without LCE. **F,** In particular, all patients identified with both LCE and high *LAMP5* expression had diffuse lytic lesions at diagnosis, or soon after. **G,** The GEP70 score was nonsignificantly higher in patients with LCE and high *LAMP5* compared with those with low *LAMP5*. **H,** The GEP5 score was significantly higher in patients with LCE and high *LAMP5* compared with those with low *LAMP5*. *, *P* < 0.05

Although the LCE subpopulation gene expression profile corresponded to risk of osteolysis and adverse prognosis, additional validation was necessary to establish how generalizable these were to other patients with LCE. Therefore, we sought additional patients with LCE in CoMMpass by filtering based on clinical light chain and M-spike data. Three criteria were used: (i) Affected light chain minus the unaffected light chain was >10 mg/dL and the M-spike was <1 g/dL at one or more visits, (ii) M-spike at diagnosis that was >1 gm/dL, and (iii) cases were excluded when the light chain increases corresponded to M-spike increases. LCE was identified in 15 of 387 (3.9%) patients with kappa/lambda/M-spike values from CoMMpass (Supplementary Fig. S8). These patients showed significantly shorter progression free survival (PFS) and OS than those without LCE (Fig. 5A and B). Despite a small sample size of 11 of 15 patients with LCE with both RNA-seq and clinical outcomes data, those with LCE and high *LAMP5* expression had a trend toward shorter PFS and OS compared with those with low *LAMP5* (Fig. 5C and D). Of the patients analyzed for LCE in CoMMpass, 187 had osteolytic information. The time to diffuse osteolytic lesions was nonsignificantly shorter in the patients with LCE compared with those without (Fig. 5E). However, patients with LCE and high *LAMP5* had uniform early presence of diffuse lytic lesions (Fig. 5F). Further showing that patients with both LCE and *LAMP5* were especially high risk, the GEP70 and GEP5 scores were relatively higher in patients with LCE with high *LAMP5* compared with those with LCE and low *LAMP5* (Fig. 5G and H). These data further support the findings from our index patient studied by scRNA-seq as being generalizable to patients with multiple myeloma that have LCE.

### Transcriptomic subpopulations are related to genetic subclones that evolve over time

**Figure 6:**
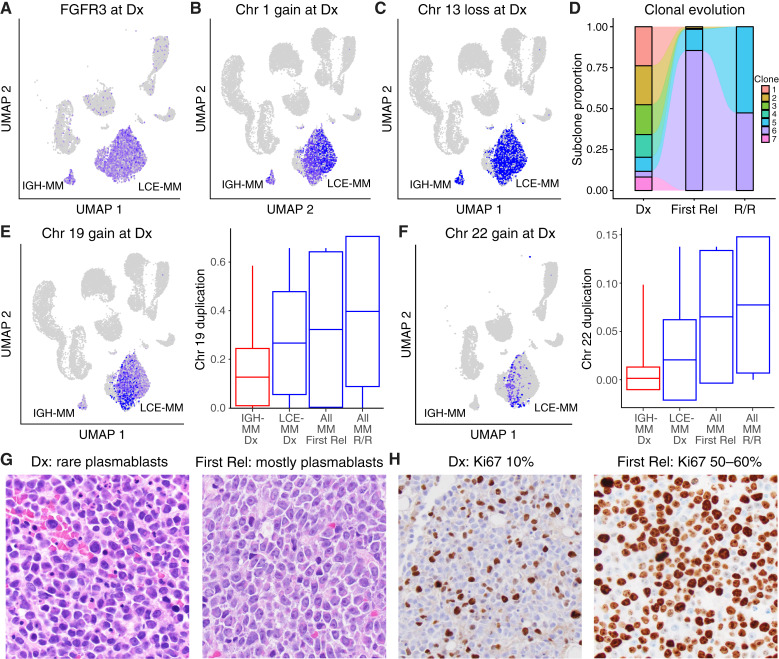
The LCE-multiple myeloma (MM) subpopulation conferred a poor prognosis and represented a genetic subclone of MM cells. **A,** FGFR3 expression distribution was high across all MM cells at diagnosis, but low or undetectable in normal cell populations, supporting clonal distribution of t(4;14) in all malignant cells. **B** and **C,** InferCNV analysis identified clonal chromosome 1 gain and chromosome 13 loss in both subpopulations, consistent with findings by FISH. **D,** Alluvial plot showing that seven subclones were present at diagnosis, five were eliminated with treatment, and two persisted across timepoints. **E,** InferCNV also identified a chromosome 19 gain that was subclonal and mostly specific to LCE-MM (left), and this increased in proportion with the disease progression (box and whiskers show median, quartiles, and 1.5 times the interquartile range). **F,** InferCNV also detected a chromosome 22 gain in a subset of LCE-MM at diagnosis (left), which increased in proportion over time. **G,** Hematoxylin and eosin staining of bone marrow samples contained some large plasmablasts at diagnosis, but at first relapse the bone marrow consisted of predominantly plasmablasts. **H,** Ki67 staining from samples at diagnosis were positive in 10% of cells, increased to 50%–60% at first relapse. Rel, relapse; R/R, relapsed/refractory timepoint.

We next sought to understand the origins of multiple myeloma subpopulations and how they may have affected the poor outcome for our patient. At diagnosis, clinical cytogenetics reported that t(4;14), gain of chromosome 1q and loss of chromosome 13 were present in all cells, and a subclonal t(8;14) was present in approximately half the cells. Consistent with t(4;14) being the initiating genetic event in this patient’s multiple myeloma, *FGFR3* was evenly overexpressed in multiple myeloma cells from both subpopulations and at each timepoint (Fig. 6A). Although no patient material was left over for genomic sequencing, we did execute infer copy number variant (inferCNV) analysis of the scRNA-seq data to further evaluate clonal versus subclonal changes (Supplementary Fig. S9A–S9C). By inferCNV, chromosome 1q gain and 13q loss were confirmed to be present in most multiple myeloma cells and in both subpopulations (Fig. 6B and C). InferCNV was evaluated across timepoints, showing that among seven distinct subclones present at diagnosis, one corresponding to LCE-multiple myeloma survived and became dominant at first relapse, and a second persisted and increased at the R/R timepoint (Fig. 6D). Similar to the *MYC* overexpression from t(8;14) detected by cytogenetics, there was a subclonal duplication of chromosome 19 that was frequent in the LCE-multiple myeloma and mostly absent in IGH-multiple myeloma (Fig. 6E). Gain of chromosome 19 was also observed by clinical chromosome analysis (Supplementary Table S1). Of note, this finding from inferred CNV correlated with the expression of the *ATF5* gene (chr 19q13.33), which was the third most upregulated gene in LCE-multiple myeloma versus IGH-multiple myeloma. No duplications or deletions were uniquely detected in IGH-multiple myeloma. These findings suggest subclonal t(4;14) and gain of chromosome 19 changes were associated with LCE-multiple myeloma subpopulation clustering.

We next sought to further validate the subclonal evolution that occurred over time in this patient. The subclonal duplication of chromosome 19 that occurred in LCE-multiple myeloma increased at timepoints 2 and 3 (Fig. 6E). We also identified a chromosome 22 duplication present in less than half of LCE-multiple myeloma cells at diagnosis, almost completely absent in IGH-multiple myeloma, then present in >90% multiple myeloma cells at first relapse and distributed in both subpopulations at the R/R timepoint (Fig. 6F). Subclone #5 was a mix of LCE-multiple myeloma and IGH-multiple myeloma, with chromosome 13 loss and chromosome 1 gain like the other subclones, but without chromosome 22 gain. Subclone #6 was all LCE-multiple myeloma and harbored the chromosome 22 gain that increased with disease recurrence. Thus, the LCE-multiple myeloma subpopulation was linked to clonal evolution through the disease course. Along with clonal evolution, phenotypic changes were also found in clinical evaluations. Hematoxylin and eosin with ki-67 stain found that bone marrow cells from diagnosis contained few plasmablasts and were 10% positive for ki-67, a higher-than-expected number for multiple myeloma (Fig. 6G and H). Then, at first relapse bone marrow cells were predominantly plasmablasts and 50% to 60% were positive for ki-67 (Fig. 6G and H). Therefore, with disease progression an increasingly prevalent plasmablastic population emerged.

### Disease progression marked by emergence of drug-resistant subpopulation

**Figure 7:**
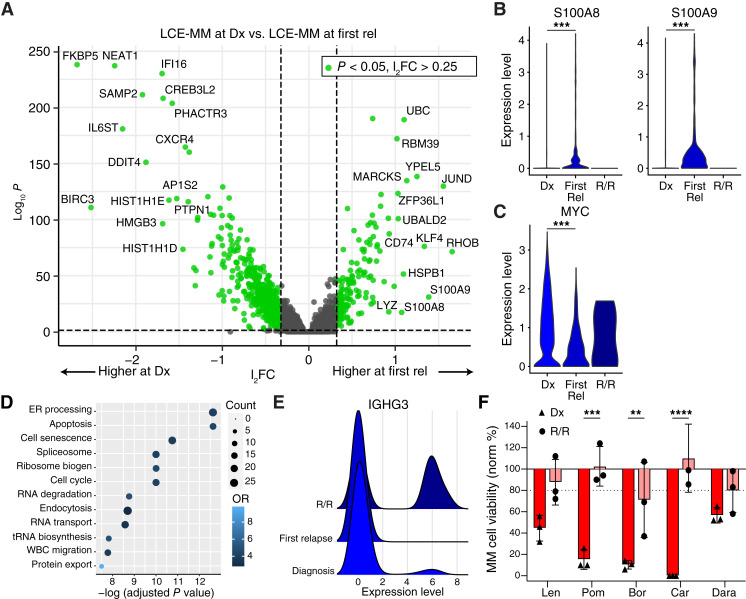
Relapse and disease progression associated with dysregulated apoptosis and increasing drug resistance. **A,** Volcano plot showing the differential gene expression between LCE-multiple myeloma (MM) cells at timepoint 1 (diagnosis) and LCE-MM cells at timepoint 2 (first relapse, with 1,042 genes higher in LCE-MM at diagnosis, 281 genes higher at first relapse, indicated by green dots for l_2_FC > 0.25, *P* < 0.05). Tested by linear regression, *n* = 4,370 cells at diagnosis, *n* = 281 cells at first relapse. **B,** Calcium-binding proteins *S100A8* and *S100A9* transcripts were increased at first relapse compared with diagnosis. **C,***MYC* was decreased at first relapse compared with diagnosis. **D,** KEGG enrichment analysis of differentially expressed genes identified several pathways including protein processing, apoptosis, and cellular senescence. **E,** MM at the R/R timepoint showed a reemergence of IGH genes. **F,** Multidrug resistance was observed *ex vivo* by My-DST at the R/R timepoint (*n* = 3, *t* test, bars show standard deviation. **, *P* < 0.01; ***, *P* < 0.001). Chr, chromosome; Dx, diagnosis; Rel, relapse; OR, odds ratio; R/R, relapsed/refractory timepoint.

To understand differential gene expression with disease progression, we compared the LCE-multiple myeloma cells at diagnosis and the LCE-multiple myeloma cells at first relapse. The refractory timepoint was not included in the differential expression analysis due to the low numbers of cells captured. Transcriptomically, 1,042 genes were higher in LCE-multiple myeloma at diagnosis (l_2_FC > 0.25, *P* < 0.05) and 281 genes were higher at first relapse (Fig. 7A; Supplementary Tables S4 and S5). Proteasome inhibitor (PI) resistance genes *S100A8* (l_2_FC = 1.07, *P* = 5.31 × 10^−18^) and *S100A9* (l_2_FC = 1.38, *P* = 1.17 × 10^−31^) were upregulated at first relapse (Fig. 7B; ref. 23). *MYC* (l_2_FC = 0.98, *P* = 3.38 × 10^−28^) was downregulated (Fig. 7C). Levels of these genes from R/R disease are shown for comparison, although the low number of multiple myeloma cells sequenced at the third timepoint limits their interpretation. KEGG enrichment analysis of differentially expressed genes identified several pathways upregulated upon relapse including protein processing, apoptosis and cell cycle (Fig. 7D). Within the apoptotic pathway, the proapoptotic genes, caspases 3, 8, and 10, were downregulated (l_2_FC = 0.52–0.59; *P* < 1 × 10^−20^) and antiapoptotic genes including *NFKBIA* were upregulated (l_2_FC = 0.57; *P* = 8.32 × 10^−17^; Supplementary Fig. S10). Thus, at first relapse, the LCE-multiple myeloma subpopulation showed evolution toward dysregulated apoptosis and drug resistance against proteasome inhibitors.

Eighteen months from diagnosis, and nine months after first relapse, our study patient developed multi-relapsed disease (timepoint 3, R/R) and further lines of treatment were ineffective. Unfortunately, there were much lower number of cells captured successfully by scRNA-seq from this timepoint, limiting interpretation. The detected R/R multiple myeloma cells were clustered across both subpopulations, and renewed expression of the IGH genes was observed (Fig. 7E). This change corresponded with a switch from light chain predominant to M-spike predominant disease (see Fig. 1C). Phenotypically, the multiple myeloma cells from the third, R/R timepoint showed significant losses of sensitivity to lenalidomide, pomalidomide, bortezomib, and carfilzomib by My-DST (Fig. 7F), consistent with that timepoint being obtained after relapsing on daratumumab, pomalidomide, and dexamethasone and progressing though carfilzomib, pomalidomide, and dexamethasone. Overall, a switch from light chain to M-spike secretion occurred, which we suspect resulted from outgrowth of a multidrug-resistant subclone, leading to the treatment-refractory disease at this timepoint.

## Discussion

Multiple myeloma is an incurable blood cancer due to enduring populations of residual cells that persist through treatment and inevitably develop drug resistance. One limitation on studies of patients with multiple myeloma is that most have not accounted for disease heterogeneity, instead distilling results to single average values for bulk multiple myeloma populations. Thus, an important next step is to understand how intrapatient heterogeneity affects drug resistance development, especially considering that multiple genetic subclones evolve as the disease progresses (6, 7). The advent of single-cell technologies enables increasingly granular evaluation of multiple myeloma, with studies so far focusing on between-patient comparison, it has been shown that transcriptomic subpopulations are common (8). Here, we saw an opportunity to interrogate intrapatient disease heterogeneity in a patient who had LCE at first relapse, making their clinical SPEP and serum free light chain values representative of divergent subclones. This proved useful for the temporal tracking of transcriptomic subpopulations of multiple myeloma cells through diagnosis, first relapse and upon development of relapsed/refractory disease to IMiDs, proteasome inhibitors and daratumumab. By this approach, we investigated the relationship between risk, osteolysis, subclonal evolution, and the development of multidrug resistance. To generalize findings from this patient, we found validating data from the MMRF CoMMpass database showing that patients with LCE and *LAMP5* gene overexpression had a high-risk gene expression, propensity to develop osteolytic bone disease and shorted survival outcomes.

scRNA-seq detected two subpopulations of multiple myeloma in our patient with LCE. The isolated expression of kappa light chain gene in LCE-multiple myeloma at diagnosis enabled tracking of this subpopulation from diagnosis using contrasting clinical measurements of kappa light chain and M-spike values. Both subpopulations were detected when M-spike and kappa light chains were high at diagnosis, but at first relapse only LCE-multiple myeloma was detected by scRNA-seq when only kappa light chain was elevated. Thus, there is compelling evidence that there were transcriptomically and functionally disparate populations of multiple myeloma, with a subpopulation present at diagnosis without LCE acting as the precursor leading to the LCE at relapse. This presents an interesting case that extends previous multiple myeloma studies finding intra-tumor mutational heterogeneity, CNA heterogeneity, and intrapatient regional tumor heterogeneity (8, 24–26). Transcriptomic subpopulations seem to be related, in part, to subclonal genetic events, as in our patient gain of chromosome 19 and 22 were inferred mostly in the LCE-multiple myeloma subpopulation. Copy number alternations can occur as drivers of progression to increasingly aggressive and treatment resistant phenotype (27, 28). This subclonal evolution seems to have led to high-risk gene expression profile, with enrichment of the GEP70 and GEP5 signatures, with notable overexpression of the potentially targetable *FABP5* (19, 20, 29). The implications of transcriptomic subpopulations are substantial for precision medicine, high-risk disease, characterization of minimal residual disease, and informing treatment decisions to deepen response. In the CoMMpass database, we found the rate of LCE to be 3.9% (*n* = 387), implicating a low but consistent rate of this phenomenon in multiple myeloma as has been reported previously (4).

Differential gene expression LCE subpopulations corresponded closely with osteolytic gene-set expression. LCE-multiple myeloma highly expressed *LAMP5*, a novel gene that has emerged from scRNA-seq data to be osteolysis promoting (Fig 2A and B; ref. 9). Increases in serum calcium of the patient in our study directly mirrored the serum light chain quantities, suggesting that osteolysis occurred to a greater extent when LCE-multiple myeloma was active (Fig. 4A). Interestingly, *LAMP5* has also been found to be upregulated in active multiple myeloma compared with smoldering multiple myeloma and thus may contribute directly to symptomatic myeloma diagnosis through development of bone lesions (30). We found the importance of the osteolytic gene expression profile generalized well in large bulk RNA-seq datasets from the MMRF and GSE24080, and in particular those patients from CoMMpass that also had LCE. Patients had a greater number of osteolytic lesions at diagnosis, as well as a greater propensity for development of diffuse lesions over time (Figs. 4F, G, 5E, and F). Interestingly, Merz and colleagues (22) also found lower expression of light chain and immunoglobulin in lytic lesions compared with bone marrow in some patients, similar to our finding that an osteolytic subpopulation in the bone marrow had lost the ability to make fully intact immunoglobulin. Together, these findings suggest a relationship between decreased immunoglobulin secretion and osteolytic activity that warrants further study.

The delineation of subpopulations of multiple myeloma within this patient allowed interrogation of their respective sensitivity or resistance to chemotherapy. Clinical treatment with bortezomib, lenalidomide, and dexamethasone reduced the burden of both multiple myeloma subpopulations, but LCE-multiple myeloma became rapidly resistant and caused early relapse before transplant. Consistent with the clinical course, *ex vivo* My-DST found multiple myeloma cells with expression pattern of LCE-multiple myeloma were already relatively resistant to IMiDs and bortezomib at diagnosis. Interestingly, KEGG gene enrichment analysis uncovered the interrelated pathways of oxidative phosphorylation and HIF-1 signaling as overrepresented in LCE-multiple myeloma and IGH-multiple myeloma, respectively. Previously, increased oxidative phosphorylation has been shown to confer resistance to proteasome inhibitor therapy (9). Within this pathway, the cyclooxygenase and NADH dehydrogenase (NDUF) gene families comprised many of the genes expressed at a higher level in LCE-multiple myeloma, also factors associated with worse survival (31–33). LCE-multiple myeloma’s high risk was corroborated by the worse prognosis of patients with similar transcriptomes in the MMRF and GSE24080 datasets.

This patient’s disease progressed from rare plasmablasts and 10% ki-67 staining to predominantly plasmablasts and >50% ki-67 staining only 9 months from diagnosis. This corresponded with the emergence of LCE-multiple myeloma as the dominant subpopulation at first relapse timepoint, when it showed dysregulation of many resistance-related genes that may have supported its survival. Interestingly, S100 genes have been found to confer resistance to proteasome inhibitors, of which the members *S100A8* and *A9* were among the genes with the greatest increase in expression in relapse versus diagnosis LCE-multiple myeloma (23). Late in the disease course, the disease switched from production of light chain to M-spike. The subclonal gain of chromosomes 19 and 22 in LCE-M increased over time to the R/R timepoint, while simultaneously heavy chain expression re-emerged. Thus, it seems that subclonal evolution was responsible for the switch back to monoclonal protein at the relapsed/refractory timepoint.

In multiple myeloma, disease heterogeneity has important ramifications for the depth of response and optimal drug combinations for patients. One implication of disease heterogeneity is that subpopulations resistant to the next line of therapy will result in a partial (incomplete) response. Confirmation of this finding would provide understanding of why short remission times and early relapses occur in some patients. A second implication is that accounting for divergent subpopulation drug sensitivity profiles may facilitate the design of better treatments and synergistic drug combinations. Proving this would unlock treatment regimens that retain better efficacy in relapsed patients. Concurrent transcriptomic and phenotypic profiling of single cells supports this effort by showing how unique multiple myeloma subpopulations within a single patient can have effects on incomplete response and improve drug combinations. Future refined study of the drug sensitivity differences of transcriptomic subpopulations may shed light on this.

## Supplementary Material

Supplemental Table 1 Clinical Characteristics of Patient #1093

Supplemental Table 2 Top 15 genes higher in LCE-MM vs IGH-MM at diagnosis.

Supplemental Table 3 Top 15 genes higher in IGH-MM vs LCE-MM at diagnosis.

Supplementary Table 4 Top 15 Genes increased in relapse vs diagnosis LCE-MM.

Supplemental Table 5 Top 15 genes increased in diagnosis vs relapsed LCE-MM.

Supplemental Figure 1 Normal Bone Marrow Cell Populations Across Timepoints.

Supplemental Figure 2 Analysis without IGH Genes Still Shows Two Subpopulations.

Supplemental Figure 3 Immunoglobulin Heavy Chain Genes were the Top Overexpressed Genes in IGH-MM.

Supplemental Figure 4 Oxidative Phosphorylation Pathway Genes Overexpressed in LCE-MM.

Supplemental Figure 5 HIF1 Signaling Pathway Genes Overexpressed in IGH-MM.

Supplemental Figure 6 Characterization of LAMP5 Effects on MM cell Prognosis, Prognosis and Viability.

Supplemental Figure 7 The IGH-MM Gene Signature Was Not Associated with Adverse Prognosis.

Supplemental Figure 8 Patients Identified from the CoMMpass Dataset with Light Chain Escape.

Supplemental Figure 9 Infer Copy Number Variant Analysis for Subclonal Tracking.

Supplemental Figure 10 Gene Expression at First Relapse Exhibiting a Dysregulation of Apoptosis.
