# Supplementary material for: Single-Cell RNA Sequencing before and after Light Chain Escape Reveals Intrapatient Multiple Myeloma Subpopulations with Divergent Osteolytic Gene Expression
Source: Cancer Res Commun. 2025 Jan 16;5(1):106–18. doi: 10.1158/2767-9764.CRC-24-0170 (PMC11737298; doi:10.1158/2767-9764.CRC-24-0170)
Supplement: Supplemental Table 1 — Clinical Characteristics of Patient #1093 [file crc-24-0170_supplemental_table_1_suppst1.pdf]

Supplemental Table 1. Clinical Characteristics of Patient #1093

Feature	Diagnosis (Research Timepoint 1)	1st Relapse (Research Timepoint 2)	2nd Relapse	Relapsed/ Refractory (Research Timepoint 3)
Karyotype	45,XY,der(1)**del(1)(p12p13)del(1)(p22p32)**,del(2)(q31q33),der(3)t(3;8)(q27;q22),der(6)t(1;6)(q12;q21),**t(8;14)(q24;q32),-13**, der(15)t(15;21)(p12;q11.2),**+19**, -21[16]/46,XY[3]	45,XY,der(1)**del(1)(p12p13)del(1)(p22p32)**,del(2)(q31q33),der(3)t(3;8)(q27;q22),der(6)t(1;6)(q12;q21),**t(8;14)(q24;q32),-13**, der(15)t(15;21)(p12;q11.2),**+19**, -21[2]/43,sl,-Y, -19[9]/45,sl,-19,+add(19)(q13.3)[4]/44,sdl2,-Y[5]	45~46,XY,der(1)**del(1)(p12p13)del(1)(p22p32)**,del(2)(q31q33),der(3)t(3;8)(q27;q22),der(6)t(1;6)(q12;q21),**t(8;14)**(q24;q32),+9,**-13**, der(15)t(15;21)(p12;q11.2), **+19**,-21[cp5]/44~45,sl,-Y, -9, add(19)(q13.3), der(19)t(9;19)(q13;q13.3)[cp4]/ 44,sl,-Y,-9, der(19)t(1;19)(q21;q13.3)[3]/44,sl,-Y,-9, der(9)t(1;9)(q21;p23),del(17)(p11.2)[3/ 70 < 3n > X,-X,-Y, der(1)del(1)(p12p13)del(1)(p22p32),del(2)(q31q33),+3, der(3)t(3;8)(q27;q22)x2, +5,+6,der(6)t(1;6)(q12;q21)x2,**t(8;14)(q24;q32)**,+9,-13, der(14)t(8;14)(q24;q32), der(15)t(15;21)(p12;q11.2), **+19**,add(19)(q13.3)x2,+20,-21,-21[2]/46,XY[2]	45,XY,+1,**del(1)(p22p34),add(1)(p12)**,add(2)(q31),add(3)(q27),der(6)t(1;6)(q16;q12**),-13**, der(15;21)(q10;q10),add(22)(q11.2)[3]/45,sl,del(11)(q14q22)[2]/45,sl,der(10)t(1;10)(q12;q22),add(16)(p12)[4]/44,sl,-Y, -add(1)(p12), add(8)(q24),der(9)t(1;9)(q12;p23),add(14)(q32), add(17)(p12**),+19[4]/79**, sdl3x2,-del(1)(p22p34), -add(3)(q27), -4,-10, -11, -12,-16,-19,-20[2]/75,sdl5, -add(3)(q27),+9,-der(9)t(1;9)(q12;p23)x2, +11,+16, -del(17)(p12)x2,-19,der(19)t(17;19)(q21;q13)x2,+20,+20,-21, -22[2]/46,XY[3]
FISH	IGH rearrangement (83.5%), 1q+/1p- (84.5%), 13q-, (97%), t(8;14) (41.8%)	t(8;14) (67%), 1q+ (56.5%), 13q- (58%)	t(4;14) (81.5%), t(8;14) (97%), 1q+ (94%), 13q- (95.5%), 17p- (26%)	t(4;14) (96%), 1q+ (94.5%), 13q- (94.0%) **
Comment	Hypodiploid count, a rearrangement of 1p, t(8;14), a derivative chromosome 3 resulting from a translocation between 3q and 8q (resulting in gain of 8q material), a derivative chromosome 6 resulting from a translocation between 1q and 6q (resulting in gain of 1q material), and loss of one copy of chromosome 13.	Four related abnormal clones in twenty metaphase cells. Notable abnormalities include a hypodiploid count, t(8;14), a derivative chromosome 6 resulting from a translocation between 1q and 6q (resulting in gain of 1q material), and loss of one copy of chromosome 13.	Presence of the IGH/FGFR3 rearrangement in this sample. The typical positive fusion pattern was observed (22.0%) as well as a variant pattern in which there was gain of an IGH signal (59.5%). These studies also demonstrate the presence of a variant IGH/MYC rearrangement with one fusion signal, two copies of MYC, and two to three copies of IGH.	Cytogenetic studies of a bone marrow aspirate specimen revealed six related abnormal clones in seventeen of twenty metaphase cells examined. Notable abnormalities include gain of multiple copies of 1q, additional material on 8q and 14q, and loss of chromosome 13.

**FISH for MYC translocation was not performed at the multi-drug refractory timepoint. (R)-ISS: (Revised) International Staging System. BM MM: Bone marrow multiple myeloma. Clinical FISH assays were enriched for plasma cells by selecting for scoring only cells with round nuclear morphology. Abnormalities also observed using scRNA data analyses are shown in bold text. It is unclear why clinical FISH for the typical myeloma translocations including t(4;14) were not tested at diagnosis, or why FISH for t(8;14) was not tested at timepoint 3.
