# Supplementary material for: Single-Cell RNA Sequencing before and after Light Chain Escape Reveals Intrapatient Multiple Myeloma Subpopulations with Divergent Osteolytic Gene Expression
Source: Cancer Res Commun. 2025 Jan 16;5(1):106–18. doi: 10.1158/2767-9764.CRC-24-0170 (PMC11737298; doi:10.1158/2767-9764.CRC-24-0170)
Supplement: Supplemental Table 2 — Top 15 genes higher in LCE-MM vs IGH-MM at diagnosis. [file crc-24-0170_supplemental_table_2_suppst2.pdf]

Supplemental Table 2: Top 15 genes higher in LCE-MM vs IGH-MM at diagnosis.

gene	Avg log_2FC	% LC-MM	% IGH-MM	p	Adjusted p
LAMP5	1.681031	0.792	0.093	3.29E-135	6.68E-131
LY6E	1.24894	0.992	0.747	1.14E-140	2.32E-136
ATF5	1.209	0.788	0.203	7.56E-87	1.54E-82
ASS1	1.200114	0.762	0.181	1.25E-92	2.55E-88
CRIP1	1.162365	0.785	0.406	2.10E-41	4.26E-37
HIST1H4C	1.094927	0.822	0.537	7.74E-23	1.57E-18
LDHB	1.076853	0.989	0.822	2.49E-101	5.07E-97
MYC	1.06094	0.732	0.388	8.01E-38	1.63E-33
FABP5	0.997482	0.769	0.285	8.60E-67	1.75E-62
EEF1B2	0.997317	0.997	0.968	5.08E-108	1.03E-103
RACK1	0.958827	0.999	0.968	3.68E-130	7.49E-126
TMC6	0.952642	0.824	0.153	5.01E-113	1.02E-108
TIMM13	0.949342	0.96	0.655	1.08E-91	2.19E-87
NPM1	0.948013	0.997	0.94	3.01E-76	6.13E-72
HMGB3	0.930161	0.821	0.441	9.18E-36	1.87E-31

*avg log2FC: average log_2 fold change, LC: light chain, IGH: immunoglobulin heavy chain, MM: multiple myeloma, %: percentage of cells with > 0 expression.
