# Supplementary material for: Single-Cell RNA Sequencing before and after Light Chain Escape Reveals Intrapatient Multiple Myeloma Subpopulations with Divergent Osteolytic Gene Expression
Source: Cancer Res Commun. 2025 Jan 16;5(1):106–18. doi: 10.1158/2767-9764.CRC-24-0170 (PMC11737298; doi:10.1158/2767-9764.CRC-24-0170)
Supplement: Supplemental Table 3 — Top 15 genes higher in IGH-MM vs LCE-MM at diagnosis. [file crc-24-0170_supplemental_table_3_suppst3.pdf]

Supplemental Table 3: Top 15 genes higher in IGH-MM vs LCE-MM at diagnosis.

gene	Avg log_2FC	% LC-MM	% IGH-MM	p	Adjusted p
IGHG3	-7.93771	0.34	1	0	0
IGHG1	-7.62728	0.127	1	0	0
IGHV3-30	-5.90659	0.208	1	0	0
IGHG4	-5.32106	0.102	0.975	0	0
SLAMF1	-1.50044	0.12	0.644	1.28E-127	2.60E-123
CCL3	-1.26037	0.25	0.32	1.35E-08	0.000274603
IGKC	-1.19915	1	1	1.18E-142	2.39E-138
IGKV1-17	-0.98874	0.981	0.979	6.35E-75	1.29E-70
FOS	-0.97906	0.741	0.726	6.13E-13	1.25E-08
TNFRSF13B	-0.93383	0.205	0.573	5.87E-82	1.19E-77
PPP1R10	-0.91529	0.907	0.893	2.44E-45	4.96E-41
CD52	-0.87427	0.825	0.851	1.98E-29	4.03E-25
FCRL2	-0.80453	0.516	0.698	2.29E-36	4.65E-32
FCRL5	-0.80192	0.898	0.925	1.13E-36	2.30E-32
DERL3	-0.7879	0.954	0.932	1.80E-24	3.66E-20

*avg log2FC: average log_2 fold change, LC: light chain, IGH: immunoglobulin heavy chain, MM: multiple myeloma, %: percentage of cells with > 0 expression.
