# Supplementary material for: Single-Cell RNA Sequencing before and after Light Chain Escape Reveals Intrapatient Multiple Myeloma Subpopulations with Divergent Osteolytic Gene Expression
Source: Cancer Res Commun. 2025 Jan 16;5(1):106–18. doi: 10.1158/2767-9764.CRC-24-0170 (PMC11737298; doi:10.1158/2767-9764.CRC-24-0170)
Supplement: Supplementary Table 4 — Top 15 Genes increased in relapse vs diagnosis LCE-MM. [file crc-24-0170_supplementary_table_4_suppst4.pdf]

Supplemental Table 4: Top 15 Genes increased in relapse vs diagnosis LCE-MM.

gene	Avg log_2FC	% Diagnosis	% Relapse	p	Adjusted p
RHOB	1.65648	0.569	0.869	2.16E-76	4.38E-72
JUND	1.55517	0.94	0.991	8.66E-135	1.76E-130
S100A9	1.38589	0.216	0.688	5.77E-36	1.17E-31
KLF4	1.33532	0.292	0.625	3.97E-81	8.07E-77
YPEL5	1.24975	0.507	0.935	2.39E-143	4.85E-139
MARCKS	1.13259	0.513	0.938	1.07E-139	2.18E-135
UBC	1.101	0.997	0.994	4.64E-194	9.44E-190
HSPB1	1.09336	0.611	0.866	1.67E-56	3.40E-52
S100A8	1.07306	0.146	0.449	2.61E-22	5.31E-18
UBALD2	1.03339	0.766	0.955	8.37E-106	1.70E-101
ZFP36L1	1.0309	0.28	0.886	2.83E-128	5.76E-124
RBM39	1.01874	0.958	0.977	4.38E-177	8.91E-173
JUN	0.98628	0.48	0.847	1.60E-45	3.25E-41
CD74	0.92917	0.799	0.974	2.49E-92	5.07E-88
LYZ	0.92403	0.09	0.384	8.31E-23	1.69E-18

*avg log2FC: average log_2 fold change
