# Supplementary material for: Single-Cell RNA Sequencing before and after Light Chain Escape Reveals Intrapatient Multiple Myeloma Subpopulations with Divergent Osteolytic Gene Expression
Source: Cancer Res Commun. 2025 Jan 16;5(1):106–18. doi: 10.1158/2767-9764.CRC-24-0170 (PMC11737298; doi:10.1158/2767-9764.CRC-24-0170)
Supplement: Supplemental Table 5 — Top 15 genes increased in diagnosis vs relapsed LCE-MM. [file crc-24-0170_supplemental_table_5_suppst5.pdf]

Supplemental Table 5: Top 15 genes increased in diagnosis vs relapsed LCE-MM.

gene	Avg log_2FC	% Diagnosis	% Relapse	p	Adjusted p
FKBP5	2.678286	0.992	0.497	0	0
BIRC3	2.517435	0.751	0.327	9.31E-116	1.89E-111
NEAT1	2.24366	0.998	0.986	3.71E-242	7.54E-238
IL6ST	2.152051	0.961	0.756	7.35E-186	1.49E-181
SMAP2	1.922226	0.973	0.722	2.75E-216	5.58E-212
DDIT4	1.881989	0.924	0.56	4.05E-156	8.23E-152
IFI16	1.695682	0.991	0.869	3.53E-235	7.17E-231
HMGB3	1.688998	0.821	0.528	2.37E-101	4.82E-97
CREB3L2	1.684193	0.975	0.827	4.22E-213	8.59E-209
HIST1H1E	1.615802	0.946	0.787	2.36E-122	4.80E-118
PHACTR3	1.578366	0.783	0.017	1.14E-208	2.32E-204
AP1S2	1.526173	0.936	0.724	9.74E-124	1.98E-119
HIST1H1D	1.454088	0.718	0.403	1.64E-78	3.33E-74
CXCR4	1.426664	0.92	0.509	1.16E-169	2.36E-165
PTPN1	1.394058	0.937	0.767	5.87E-121	1.19E-116

*avg log2FC: average log_2 fold change
