# Supplementary material for: Single-Cell RNA Sequencing before and after Light Chain Escape Reveals Intrapatient Multiple Myeloma Subpopulations with Divergent Osteolytic Gene Expression
Source: Cancer Res Commun. 2025 Jan 16;5(1):106–18. doi: 10.1158/2767-9764.CRC-24-0170 (PMC11737298; doi:10.1158/2767-9764.CRC-24-0170)
Supplement: Supplemental Figure 1 — Normal Bone Marrow Cell Populations Across Timepoints. [file crc-24-0170_supplemental_figure_1_suppsf1.pdf]

Supplemental Figure 1. Normal Bone Marrow Cell Populations Across Timepoints.

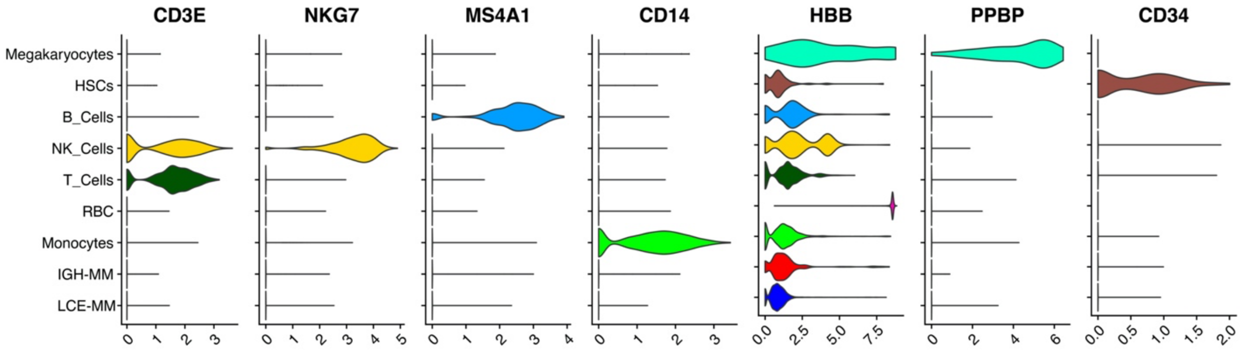

From UMAP cluster analysis, cell populations were designated by gene expression. Hallmark genes examples are shown. The T-cell cluster expressed CD3E. NK cells expressed CD3E and NKG7. Monocytes expressed CD14. Progenitors expressed CD34. Megakaryocytes expressed PPBP. RBCs express extremely high HBB in comparison to other populations.
