# Supplementary material for: Single-Cell RNA Sequencing before and after Light Chain Escape Reveals Intrapatient Multiple Myeloma Subpopulations with Divergent Osteolytic Gene Expression
Source: Cancer Res Commun. 2025 Jan 16;5(1):106–18. doi: 10.1158/2767-9764.CRC-24-0170 (PMC11737298; doi:10.1158/2767-9764.CRC-24-0170)
Supplement: Supplemental Figure 2 — Analysis without IGH Genes Still Shows Two Subpopulations. [file crc-24-0170_supplemental_figure_2_suppsf2.pdf]

Supplemental Figure 2. Analysis without IGH Genes Still Shows Two Subpopulations.

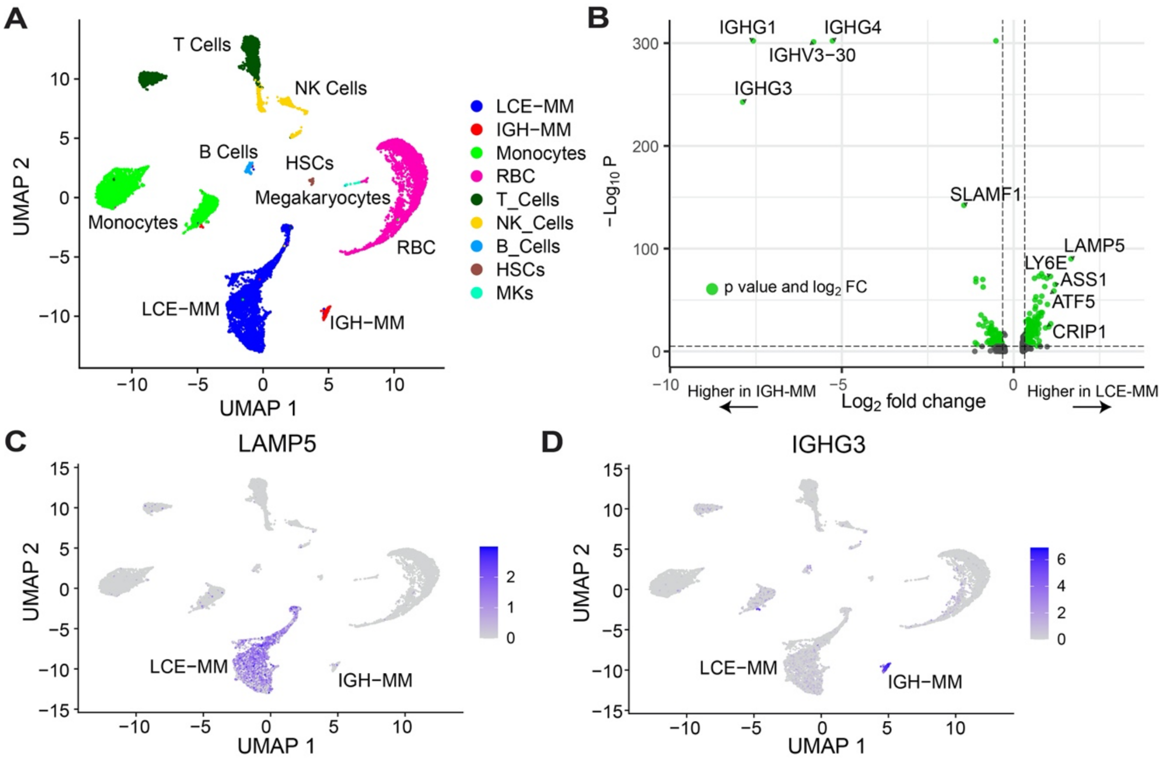

(A) UMAP clustering analysis of serial bone marrow biopsy samples from patient 1093 showed two distinct subpopulations of MM cells (LCE-MM and IGH-MM). (B) LAMP5 remained the top overexpressed gene in LCE-MM and IGH genes topped IGH-MM. (C) UMAP showing LAMP5 overexpression was specific to the LCE-MM subpopulation. (D) IGHG3 expression was still restricted to IGH-MM even when IGH mRNAs were removed from the UMAP analysis.
