# Supplementary material for: Single-Cell RNA Sequencing before and after Light Chain Escape Reveals Intrapatient Multiple Myeloma Subpopulations with Divergent Osteolytic Gene Expression
Source: Cancer Res Commun. 2025 Jan 16;5(1):106–18. doi: 10.1158/2767-9764.CRC-24-0170 (PMC11737298; doi:10.1158/2767-9764.CRC-24-0170)
Supplement: Supplemental Figure 3 — Immunoglobulin Heavy Chain Genes were the Top Overexpressed Genes in IGH-MM. [file crc-24-0170_supplemental_figure_3_suppsf3.pdf]

Supplemental Figure 3. Immunoglobulin Heavy Chain Genes were the Top Overexpressed Genes in IGH-MM.

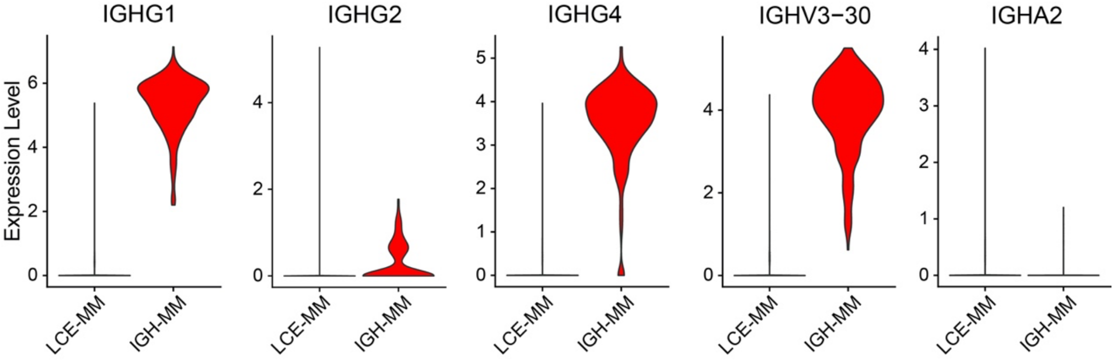

Differential expression analysis of the top overexpressed genes between subpopulations and subpopulations revealed drastic overexpression of IGH genes in the IGH-MM subpopulation and complete loss of expression in the LCE-MM subpopulation. The log2 fold-change values for IGH-MM compared to LCE-MM ranged from -5.32 to -7.94 ($p < 1 \times 10^{-150}$). By comparison, IGA was not expressed in either subpopulation.
