# Supplementary material for: Single-Cell RNA Sequencing before and after Light Chain Escape Reveals Intrapatient Multiple Myeloma Subpopulations with Divergent Osteolytic Gene Expression
Source: Cancer Res Commun. 2025 Jan 16;5(1):106–18. doi: 10.1158/2767-9764.CRC-24-0170 (PMC11737298; doi:10.1158/2767-9764.CRC-24-0170)
Supplement: Supplemental Figure 4 — Oxidative Phosphorylation Pathway Genes Overexpressed in LCE-MM. [file crc-24-0170_supplemental_figure_4_suppsf4.pdf]

Supplemental Figure 4. Oxidative Phosphorylation Pathway Genes Overexpressed in LCE-MM.

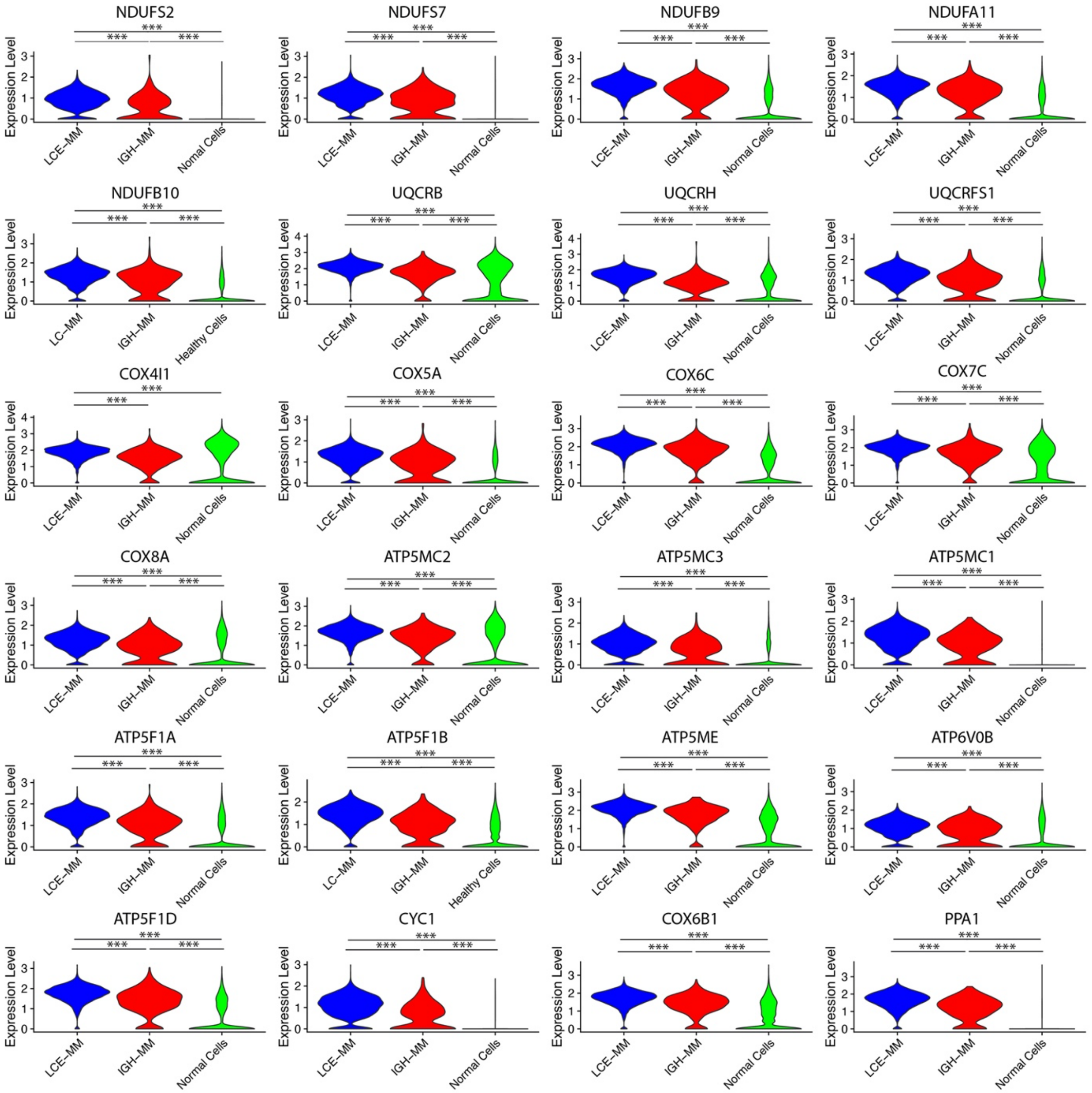

Twenty-four genes from the oxidative phosphorylation pathway were significantly higher in LCE-MM than in IGH-MM and normal cells. These included 6 genes in the COX family and 5 in the NDUF family. All but one of the genes were also expressed significantly higher in IGH-MM compared to normal cells. Statistical comparisons were made by linear regression.
