# Supplementary material for: Single-Cell RNA Sequencing before and after Light Chain Escape Reveals Intrapatient Multiple Myeloma Subpopulations with Divergent Osteolytic Gene Expression
Source: Cancer Res Commun. 2025 Jan 16;5(1):106–18. doi: 10.1158/2767-9764.CRC-24-0170 (PMC11737298; doi:10.1158/2767-9764.CRC-24-0170)
Supplement: Supplemental Figure 5 — HIF1 Signaling Pathway Genes Overexpressed in IGH-MM. [file crc-24-0170_supplemental_figure_5_suppsf5.pdf]

Supplemental Figure 5. HIF1 Signaling Pathway Genes Overexpressed in IGH-MM.

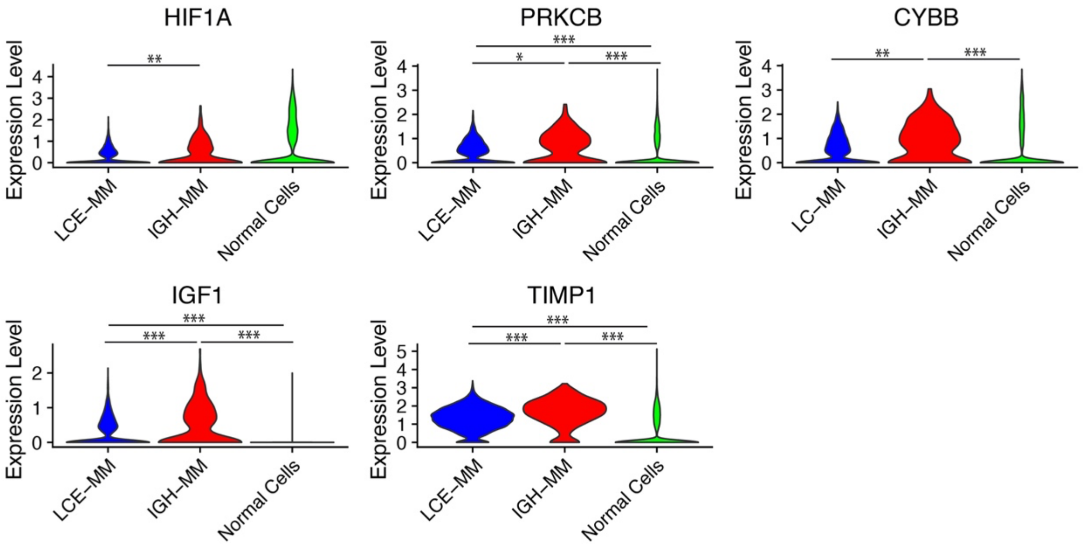

Five genes from the HIF1 signaling pathway were upregulated in IGH-MM compared to LCE-MM including IGF1 and HIF1A. Three out of the five genes (adjusted p-values: CYBB-1.91e-2, IGF1-4.8e-98, TIMP1-5.02e-111) were also expressed significantly higher in IGH-MM compared to normal (healthy cells). Statistical comparisons were done by logistic regression.
