# Supplementary material for: Single-Cell RNA Sequencing before and after Light Chain Escape Reveals Intrapatient Multiple Myeloma Subpopulations with Divergent Osteolytic Gene Expression
Source: Cancer Res Commun. 2025 Jan 16;5(1):106–18. doi: 10.1158/2767-9764.CRC-24-0170 (PMC11737298; doi:10.1158/2767-9764.CRC-24-0170)
Supplement: Supplemental Figure 6 — Characterization of LAMP5 Effects on MM cell Prognosis, Prognosis and Viability. [file crc-24-0170_supplemental_figure_6_suppsf6.pdf]

Supplemental Figure 6. Characterization of LAMP5 Effects on MM cell Prognosis, Prognosis and Viability.

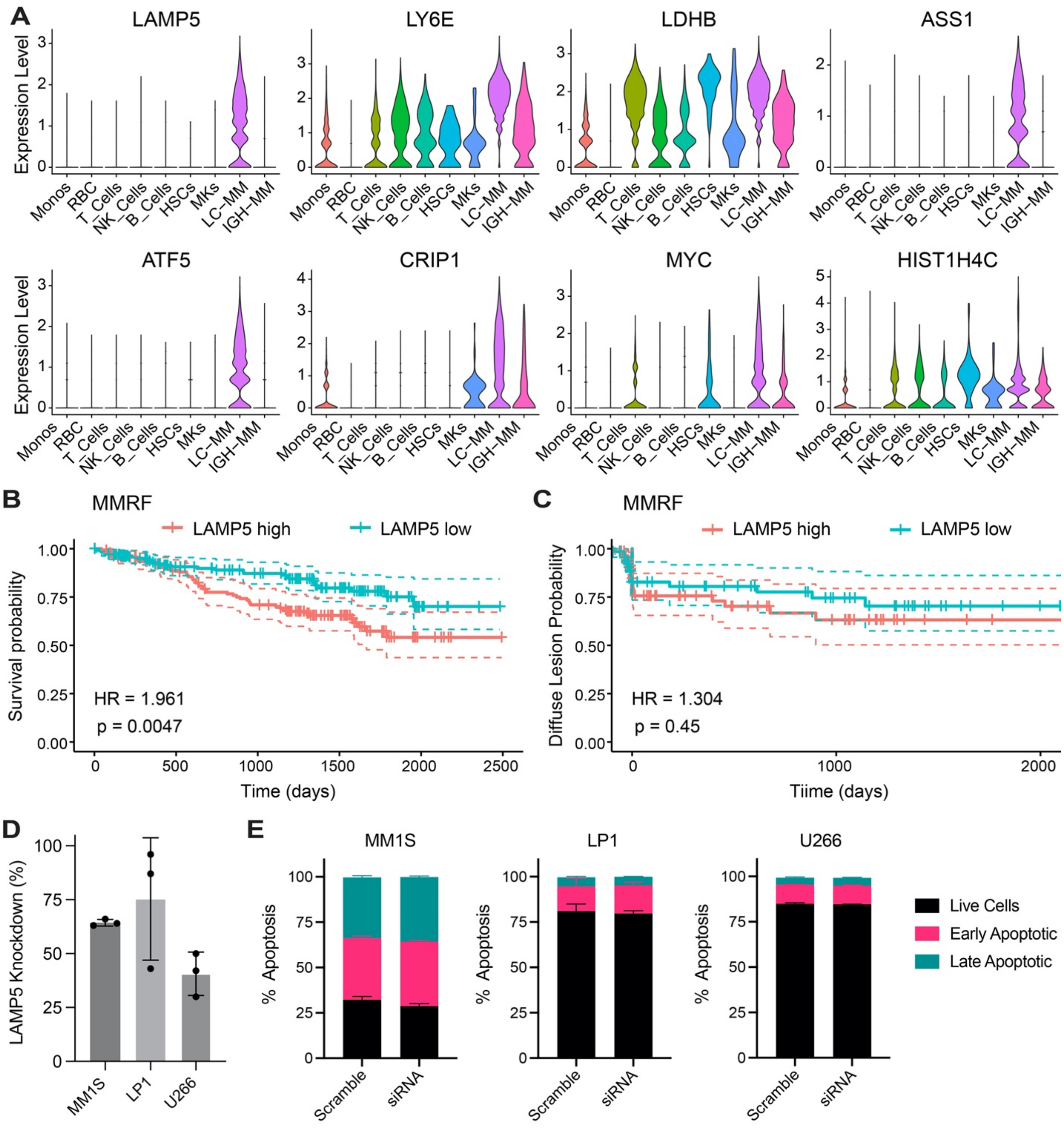

(A) Cell Type Specific Expression of LCE-MM Overexpressed Geneset. Eight genes were overexpressed in LCE-MM compared to IGH-MM by thresholds of l_2FC > 1.0 and adjusted $p < 0.05$. These genes were also expressed specifically in LCE-MM cells compared to normal cell populations. (B) In the MMRF dataset patients with high LAMP5 transcript enrichment score had worse overall survival than those with low LAMP5 enrichment. (D) Percent LAMP5 knockdown in MM cell lines 48 hrs post siRNA electroporation. (E) Inhibition of LAMP5 gene expression did not change MM cell line viability as determined by annexin DAPI staining.
