# Supplementary material for: Single-Cell RNA Sequencing before and after Light Chain Escape Reveals Intrapatient Multiple Myeloma Subpopulations with Divergent Osteolytic Gene Expression
Source: Cancer Res Commun. 2025 Jan 16;5(1):106–18. doi: 10.1158/2767-9764.CRC-24-0170 (PMC11737298; doi:10.1158/2767-9764.CRC-24-0170)
Supplement: Supplemental Figure 7 — The IGH-MM Gene Signature Was Not Associated with Adverse Prognosis. [file crc-24-0170_supplemental_figure_7_suppsf7.pdf]

Supplemental Figure 7. The IGH-MM Gene Signature Was Not Associated with Adverse Prognosis.

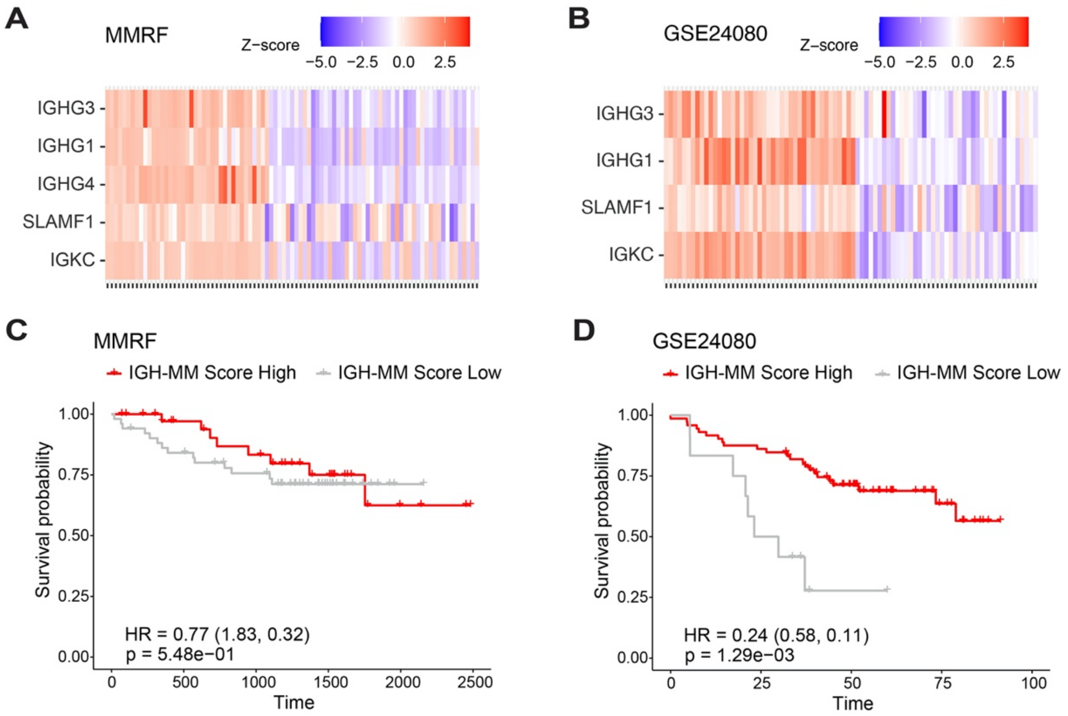

From the MMRF and GSE24080 datasets 325 and 254 samples were from IgG predominant MM, of which 38 and 43 samples had high IGH-MM gene-set scores, and 51 and 41 samples had low MM2 gene-set scores in MMRF and GSE24080 respectively. (A-B) The gene's comprising the signatures compared in MMRF and GSE24080 are shown. (C-D) The IGH-MM gene set was not associated with adverse prognosis. In the MMRF and GSE24080 datasets with non-significant HR of 0.77 in the MMRF (CI = 1.8-0.3, p = 5.48 x 10^{-1}) and significant HR of 0.25 in GSE24080 (CI = 0.6-0.1, p = 1.29 x 10^{-3}).
