# Supplementary material for: Single-Cell RNA Sequencing before and after Light Chain Escape Reveals Intrapatient Multiple Myeloma Subpopulations with Divergent Osteolytic Gene Expression
Source: Cancer Res Commun. 2025 Jan 16;5(1):106–18. doi: 10.1158/2767-9764.CRC-24-0170 (PMC11737298; doi:10.1158/2767-9764.CRC-24-0170)
Supplement: Supplemental Figure 8 — Patients Identified from the CoMMpass Dataset with Light Chain Escape. [file crc-24-0170_supplemental_figure_8_suppsf8.pdf]

Supplemental Figure 8. Patients Identified from the CoMMpass Dataset with Light Chain Escape.

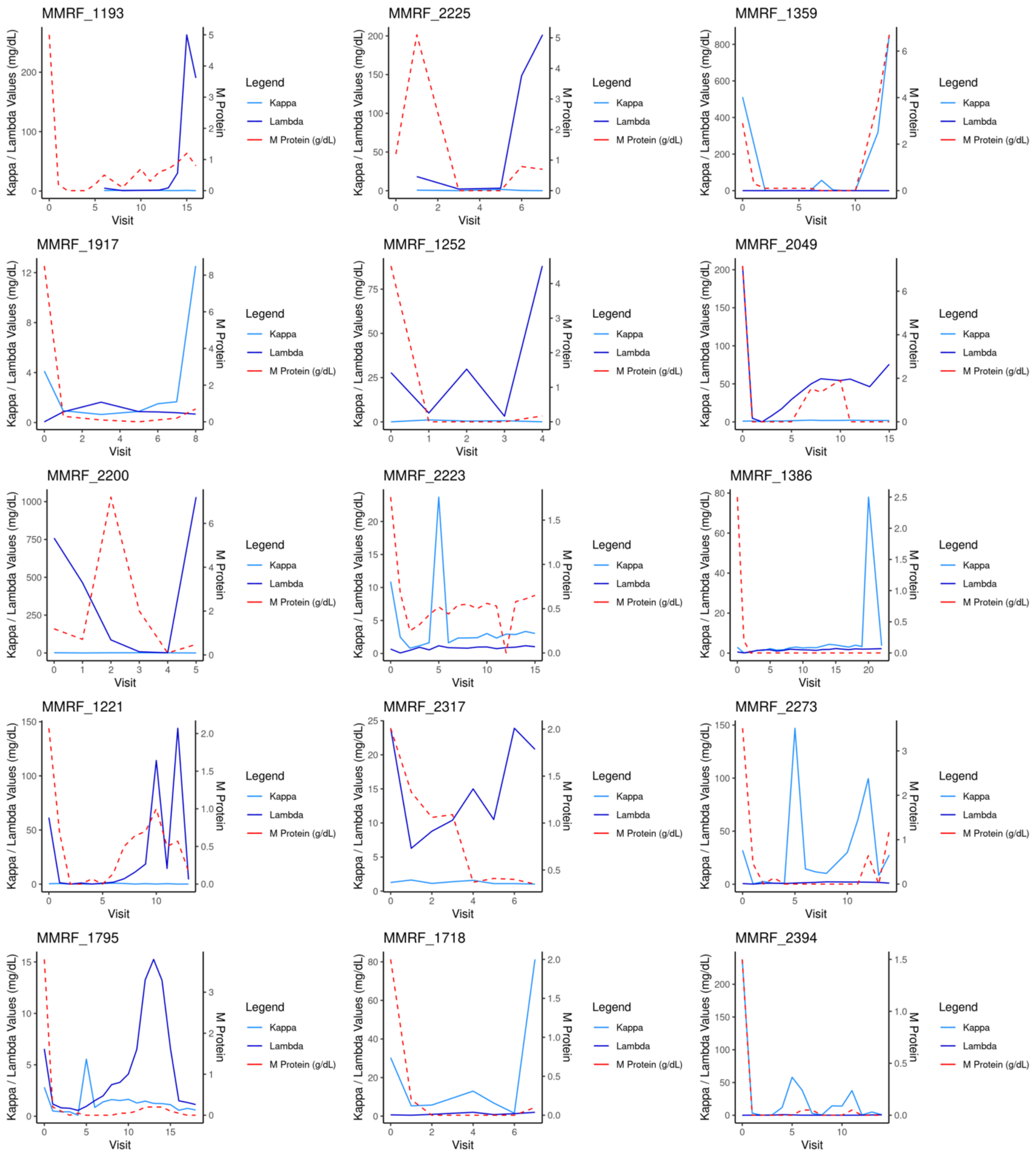

Patients with LCE were identified from the MMRF CoMMpass dataset based on having at least one timepoint when the absolute affected light chain minus the unaffected light chain was > 10 mg/dl while the M-protein was < 1 g/dL. Samples were only included if the M-protein at diagnosis was >1 gm/dl, to exclude patients with light chain only disease at diagnosis. We also excluded cases when the light chain increases corresponded to parallel increases in the M-protein.
