# Supplementary material for: Single-Cell RNA Sequencing before and after Light Chain Escape Reveals Intrapatient Multiple Myeloma Subpopulations with Divergent Osteolytic Gene Expression
Source: Cancer Res Commun. 2025 Jan 16;5(1):106–18. doi: 10.1158/2767-9764.CRC-24-0170 (PMC11737298; doi:10.1158/2767-9764.CRC-24-0170)
Supplement: Supplemental Figure 9 — Infer Copy Number Variant Analysis for Subclonal Tracking. [file crc-24-0170_supplemental_figure_9_suppsf9.pdf]

Supplemental Figure 9. Infer Copy Number Variant Analysis for Subclonal Tracking.

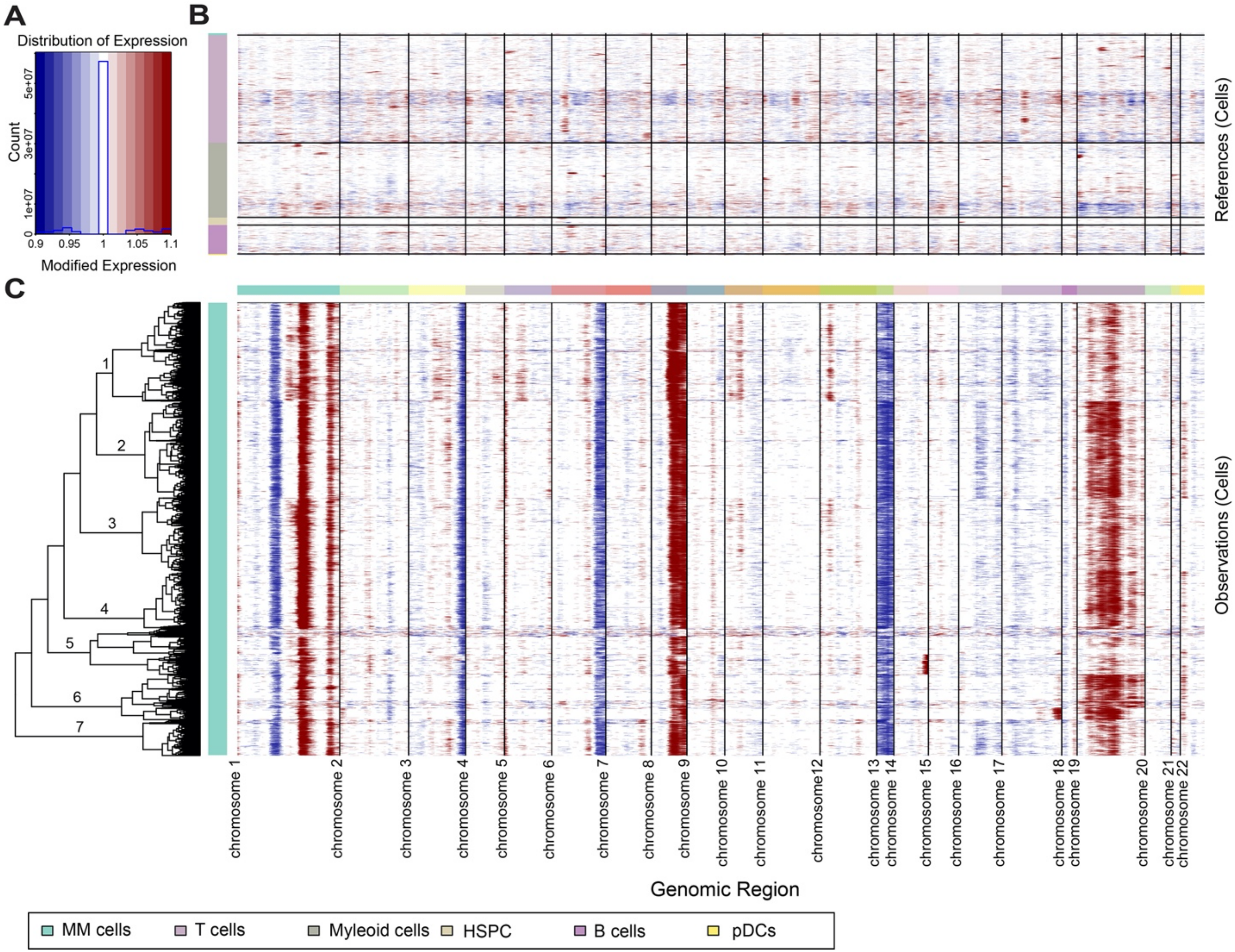

InferCNV clustering of all cells in scRNAseq experiment. (A-B) Reference cells used to infer CNVs in MM cells. (C) inferred CNVs in MM cells organized by hierarchical clustering.
