# Supplementary material for: Single-Cell RNA Sequencing before and after Light Chain Escape Reveals Intrapatient Multiple Myeloma Subpopulations with Divergent Osteolytic Gene Expression
Source: Cancer Res Commun. 2025 Jan 16;5(1):106–18. doi: 10.1158/2767-9764.CRC-24-0170 (PMC11737298; doi:10.1158/2767-9764.CRC-24-0170)
Supplement: Supplemental Figure 10 — Gene Expression at First Relapse Exhibiting a Dysregulation of Apoptosis. [file crc-24-0170_supplemental_figure_10_suppsf10.pdf]

Supplemental Figure 10. Gene Expression at First Relapse Exhibiting a Dysregulation of Apoptosis.

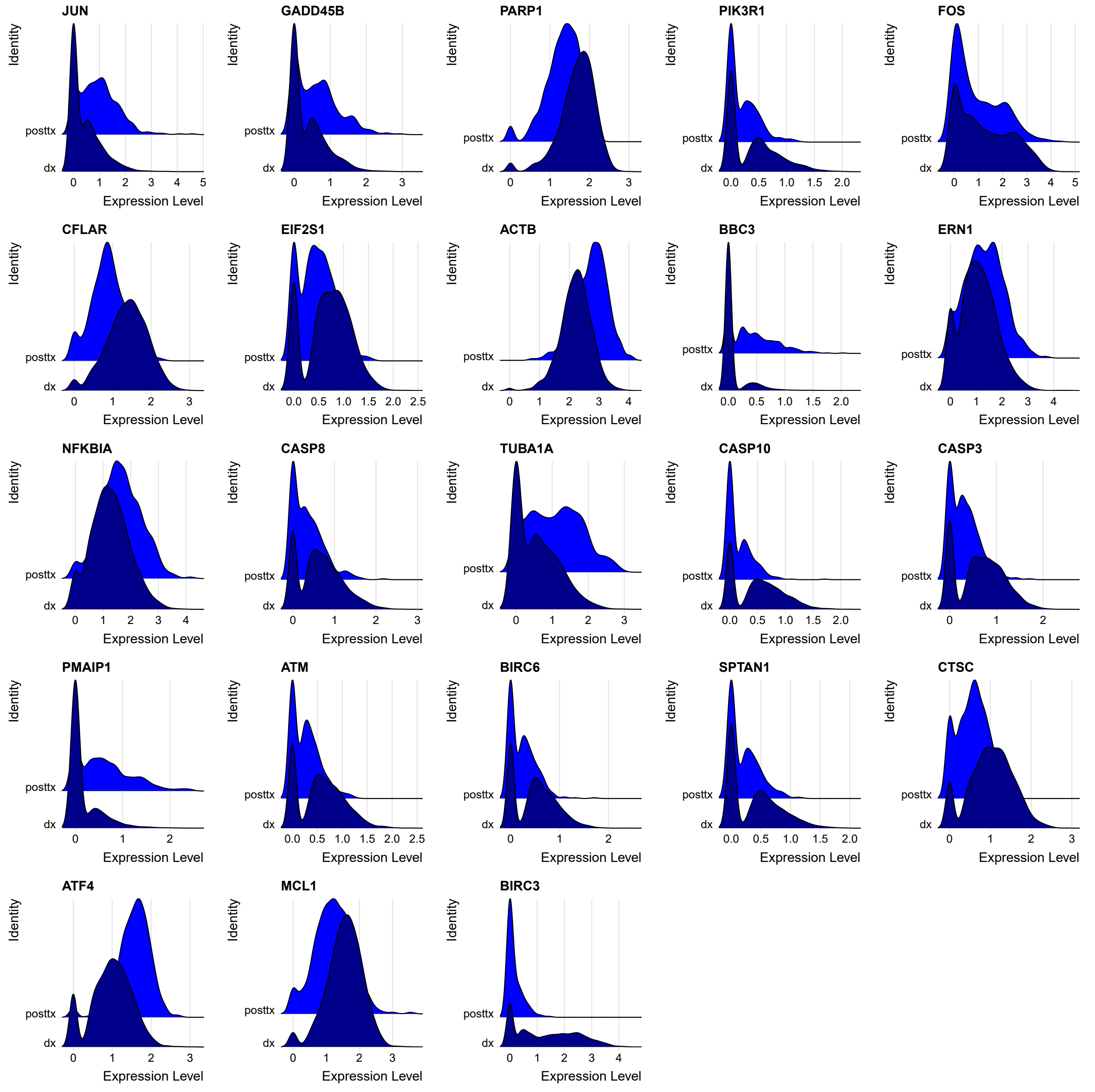

Key genes are shown that were involved in apoptosis and dysregulated in the LCE-MM subpopulation between diagnosis and first relapse. Pro-apoptotic genes including caspases 3, 8, and 10, were downregulated at relapse ($l_2FC = 0.52$-0.59, $p < 1 \times 10^{-20}$). Anti-apoptotic genes including NFKBIA were upregulated ($l2FC = 0.57$, $p = 8.32 \times 10^{-17}$) at relapse.
